# Stochastic fluctuations promote ordered pattern formation of cells in the Notch-Delta signaling pathway

**DOI:** 10.1371/journal.pcbi.1010306

**Published:** 2022-07-21

**Authors:** Madeline Galbraith, Federico Bocci, José N. Onuchic

**Affiliations:** 1 Center for Theoretical Biological Physics, Rice University, Houston, Texas, United States of America; 2 Department of Physics and Astronomy, Rice University, Houston, Texas, United States of America; 3 NSF-Simons Center for Multiscale Cell Fate research, University of California Irvine, California, United States of America; 4 Department of Chemistry, Rice University, Houston, Texas, United States of America; 5 Department of Biosciences, Rice University, Houston, Texas, United States of America; Pázmány Péter Catholic University: Pazmany Peter Katolikus Egyetem, HUNGARY

## Abstract

The Notch-Delta signaling pathway mediates cell differentiation implicated in many regulatory processes including spatiotemporal patterning in tissues by promoting alternate cell fates between neighboring cells. At the multicellular level, this "lateral inhibition” principle leads to checkerboard patterns with alternation of Sender and Receiver cells. While it is well known that stochasticity modulates cell fate specification, little is known about how stochastic fluctuations at the cellular level propagate during multicell pattern formation. Here, we model stochastic fluctuations in the Notch-Delta pathway in the presence of two different noise types–shot and white–for a multicell system. Our results show that intermediate fluctuations reduce disorder and guide the multicell lattice toward checkerboard-like patterns. By further analyzing cell fate transition events, we demonstrate that intermediate noise amplitudes provide enough perturbation to facilitate “proofreading” of disordered patterns and cause cells to switch to the correct ordered state (Sender surrounded by Receivers, and vice versa). Conversely, high noise can override environmental signals coming from neighboring cells and lead to switching between ordered and disordered patterns. Therefore, in analogy with spin glass systems, intermediate noise levels allow the multicell Notch system to escape frustrated patterns and relax towards the lower energy checkerboard pattern while at large noise levels the system is unable to find this ordered base of attraction.

## Author summary

The Notch pathway is involved in many biological processes and is known to form precise spatial patterns alternating Sender and Receiver cell states. Quantifying the implications of stochastic fluctuations provided insight that patterns formed in Notch-mediated pathways must follow a predetermined path towards checkerboard or exist in a noisy environment which promotes order through error correction.

We model Notch pattern formation stochastically and analyze the spatiotemporal dynamics. Our results show multicellular systems equilibrate towards ordered systems, but mistakes in the initial lattice propagate causing the systems to relax into frustrated systems. Only through existing in a noisy environment are the systems able to relax into the checkerboard pattern. Analyzing the temporal dynamics confirms, in environments with intermediate noise, the “incorrect” cells (Sender in a Sender environment, and vice versa) can be flipped to the correct state (Sender in a Receiver environment, and vice versa). Comparing with the spin glass energy landscape, we suggest the multicellular model follows a rugged landscape to form patterns with stochastic fluctuations required to enforce order throughout the system.

## Introduction

During many developmental and physiological processes, cells integrate information from their neighbors and the local microenvironment to attain precise patterns in space and time by communicating through several signaling pathways. Notch is an evolutionarily conserved cell-cell signaling pathway integral to cell differentiation in a range of biological processes including angiogenesis [1], neurogenesis [2], embryogenesis [3], and vein boundary formation of the fruit fly wing [4]. While the main components of the pathway are well-conserved, the spatial patterning operates under different conditions across biological systems. While several developmental systems including fruit fly wing formation [4], somite segmentation [5] or angiogenesis [1] exhibit precise spatio-temporal signals, pathological settings, including cancer, feature more heterogeneous and potentially conflicting signaling cues [6,7]. These observations underscore how single cell fate decision, intercellular communication, and even initial conditions are integrated together to achieve different spatial patterns.

**Fig 1:**
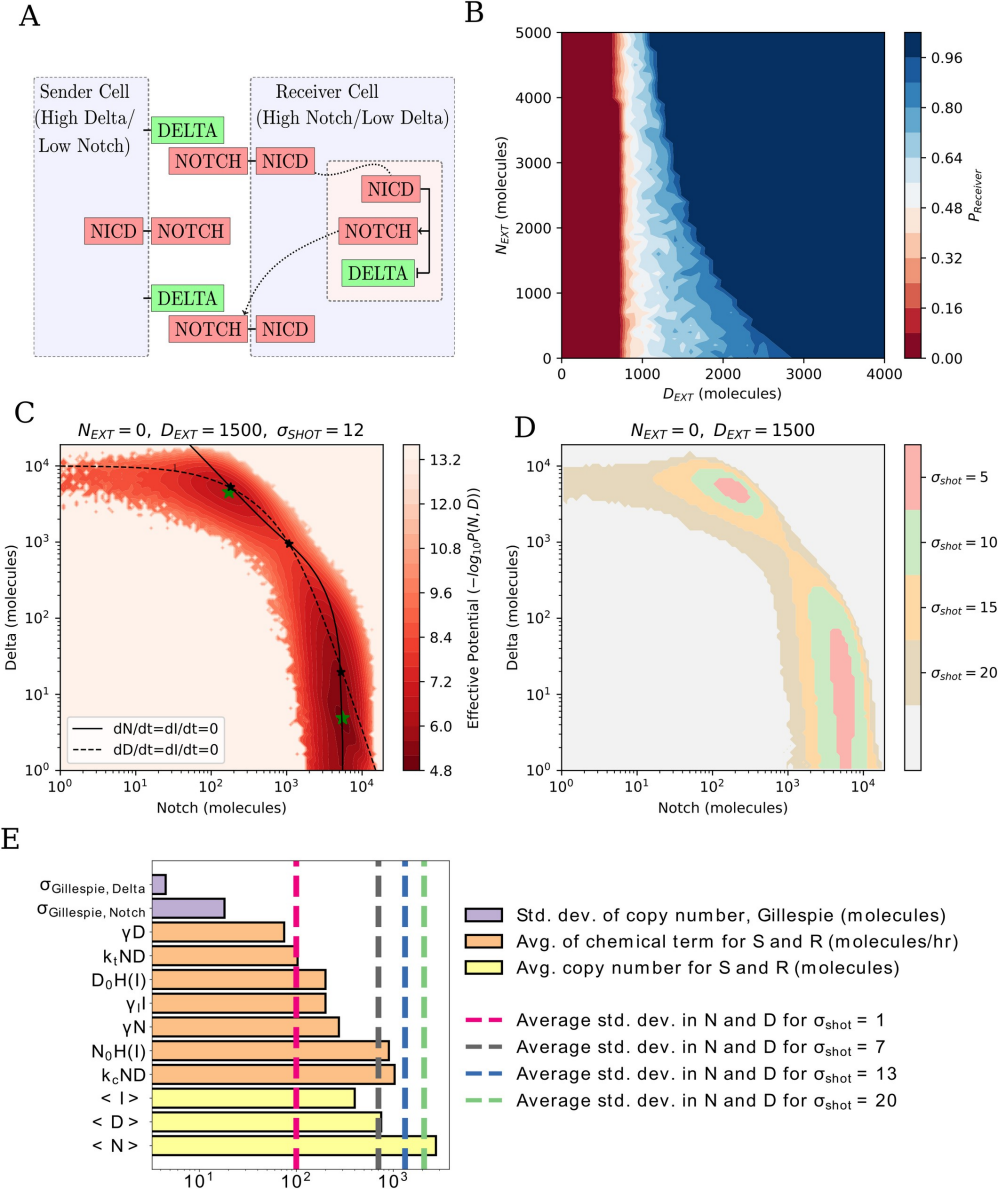
Effect of noise in the Notch-Delta single cell model. **(a)** Schematic of the Notch-Delta circuit. The Delta ligand of the Sender cell binds to the Notch transmembrane receptor of the Receiver cell. Upon binding, the Notch Intracellular Domain (NICD) translocates to the cell nucleus (pink shading), where it upregulates the expression of Notch while inhibiting the expression of Delta. **(b)** Phase diagram of the deterministic single cell model as a function of external Delta ligands (D_EXT_) and Notch receptors (N_EXT_). Color scale indicates the probability to attain a Receiver state. To estimate the Receiver state probability, n = 1000 independent trajectories starting from randomized initial conditions were solved for each (N_EXT,_ D_EXT_) combination. In the white region where the red shifts to blue, the system is bistable and can be in either the Sender or Receiver state. **(c)** Pseudopotential landscape for a case of shot noise (σ_shot_ = 12, N_EXT_ = 0, D_EXT_ = 1500). Continuous and dashed black lines depict the nullclines of the corresponding deterministic model, while black starred dots highlight the stable fixed points of the corresponding deterministic model. Green starred points locate the minima of the landscape. **(d)** Heatmap showing the extension of the Sender and Receiver minima as a function of shot noise for (N_EXT_ = 0, D_EXT_ = 1500). The areas of the two states are defined as the regions where the pseudopotential is increased by at most a unit from its value in the minimum. **(e)** Comparison of the standard deviations of Notch and Delta with different shot noise levels (dashed pink, grey, blue, and green lines representing std. dev. of N and D for σ_shot_ = 1, 7, 13, and 20, respectively) against fluctuations in Gillespie simulations (purple bars), chemical terms in the Notch circuit (orange terms), and copy numbers of Notch and Delta (yellow bars). Chemical rate amplitudes and copy numbers are averaged over Sender and Receiver. For the Gillespie and shot noise simulations, the one cell sender has N_EXT_ = 5000, D_EXT_ = 0 while the Receiver cell has N_EXT_ = 500, D_EXT_ = 1500.

At its core, Notch-Delta signaling operates as a two-cell toggle switch that leads to opposite states between neighboring cells [8]. The signaling activates when the Notch transmembrane receptor binds to the Delta transmembrane ligand of a neighboring cell and the Notch Intracellular Domain (NICD) is cleaved from the Notch receptor (Fig 1A). Once cleaved, the NICD is transported to the cell nucleus where it upregulates Notch and downregulates Delta [9–11]. This double negative feedback loop leads to opposite fates between a "Sender” cell (high Delta, low Notch) and a “Receiver” cell (low Delta, high Notch) [12]. In addition, if the Notch transmembrane receptor binds to the Delta ligand of the same cell, both Notch and Delta are degraded (cis-inhibition) [13,14].

When generalized to a multicellular scenario, lateral inhibition leads to patterns with alternating cell states, such as in the bristle patterning of the fruit fly [4,15]. The small differences between initial values of Notch and Delta in neighboring cells are amplified by the Notch-Delta negative feedback to generate precise patterns [13,15,16]. While Notch-Delta lateral inhibition is generally accepted as a qualitative model of alternate cell patterning, the precise mechanism by which cells can enforce robust patterning in a noisy cellular environment remains poorly understood. While several theoretical frameworks have been proposed to model Notch-Delta lateral inhibition, multicellular pattern formation has mostly been studied with deterministic models [12,13,15–19], despite the importance of noise in the Notch-Delta pathway in creating stable pattern formation [20–22].

In biochemical signaling networks, stochasticity is often present in the form of either thermal and small number fluctuations during transcription and protein binding (intrinsic noise) or cell-to-cell variability due to external signals in the local microenvironment (extrinsic noise) [23–26]. These sources of stochasticity can be incorporated in models of regulatory dynamics and cell-cell signaling utilizing stochastic differential equations. Different models of fluctuations have been previously employed to study gene regulatory networks. White noise represents perhaps the most general framework to introduce fluctuations and can generically represent fluctuating levels of external signals or even inhomogeneity in the diffusion of a signaling gradient [19,27–29]. Moreover, shot noise depends on protein concentration and is therefore used to model binding/unbinding events and transcriptional bursting [25,29–31]. It has been suggested that stochastic fluctuations can break the symmetry between initially similar cells with comparable levels of Notch and Delta, thus promoting opposite fates via lateral-inhibition [32]. Further, de Back and collaborators implemented noise in a system containing the Notch pathway, allowing for modulation of the system and symmetry breaking independent of noise source [27]. However, a study of both bistable and tristable gene regulatory networks by Lu and collaborators showed that white and shot noise have different effects on the stability of multistable genetic switches [29]. Lu comments that fluctuations in cells may be in an intermediate regime between white and shot noise [29]. Additionally, the importance of spatio-temporal noise in pattern formation was suggested in a Landau theory of lateral inhibition with additive white noise fluctuations [28] and a cellular automata model of lateral inhibition which included spatial noise and temporal noise during cell state updates as probabilities that a cell will escape inhibition or fail to signal its neighbors [33]. Despite these promising initial steps, the implications of white and shot noise on Notch-driven multicell signaling and patterning have not yet been quantified.

Here, we study the spatiotemporal dynamics of a multicellular Notch-Delta lattice model under the effect of stochastic fluctuations. First, we show that white and shot noise have profoundly different effects on the pseudopotential landscape of a single cell interacting with a fixed extracellular environment. While white noise tends to merge the Sender and Receiver states, shot noise more effectively maintains the bistability between states. By generalizing the model to a multicellular scenario, we quantify the robustness of the checkerboard pattern and further show that Notch-Delta signaling maximizes patterning order when operating at an intermediate noise level. While low to intermediate noise supports order by flipping incorrect cell states, high noise can potentially flip correct cell states. These results further suggest an interesting parallel between Notch multicellular patterning and the navigation of the energy landscape of spin glass models. Overall, our analysis provides new mechanistic insights into the spatio-temporal patterning driven by Notch-Delta signaling and demonstrates how precise ordering is achieved in the noisy physiological environment.

## Results

### Generalizing the Notch-Delta switch

To study the role of noise in Notch-Delta signaling dynamics, we first consider the simplified case of a single cell that is exposed to fixed levels of Delta ligands (D_EXT_) and Notch receptors (N_EXT_) that bind to cellular Notch and Delta. These external signals represent the effect of neighboring cells in Sender or Receiver states. Therefore, the single cell model can be interpreted as a mean field approximation of a multicellular lattice model.

Several models have been proposed to model the Notch-Delta switch [13,15,16,18,34,35]. In particular, to build the stochastic single cell model, we generalize the Notch-Delta switch developed by Boareto and collaborators [12] to include stochastic fluctuations on the Notch receptor and Delta ligand to account for the multicell environment. The temporal dynamics of Notch (N), Delta (D) and Notch Intracellular domain (NICD or I) copy numbers in a cell is modeled with coupled stochastic differential equations:

$$\frac{dN}{dt}=N_{0}H^{S}(I,I_{0},n_{I},\lambda_{N})-k_{c}ND-k_{T}ND_{EXT}-\gamma N+G_{N}(\sigma )dW(t),$$ (1)

$$\frac{dD}{dt}=D_{0}H^{S}(I,I_{0},n_{I},\lambda_{D})-k_{c}DN-k_{T}DN_{EXT}-\gamma D+G_{D}(\sigma )dW(t),$$ (2)

$$\frac{dI}{dt}=k_{T}ND_{EXT}-\gamma_{I}I.$$ (3)

The model includes protein production, degradation, chemical binding between Notch and Delta, release of NICD, transcriptional regulation by NICD on Notch and Delta, and stochastic fluctuations (parameters values are presented in Table A in S1 Text. Details of the model are expanded upon in S1A Text). Notch and Delta are produced with basal production rate constants N_0_ and D_0_, which are further modulated by the transcriptional activation or inhibition by NICD that activates Notch and inhibits Delta, respectively. This modulation is modeled with the shifted Hill function [36]:


$$H^{S}(I,I_{0},n,\lambda )=\lambda +\frac{1-\lambda }{1+{\left(I/I_{0}\right)}^{n}}.$$ (4)

Once the amount of NICD in the cell (I) is greater than the threshold of NICD (I_0_), the shifted Hill function is saturated, and Notch/Delta are activated/inhibited. This magnitude of upregulation or downregulation is represented by the foldchange (λ), while the sensitivity to changes in NICD levels is represented by the Hill coefficient n.

Cellular Notch receptors and Delta ligands can bind to other exogenous ligands and receptors (D_EXT_ and N_EXT_) with rate constant k_t_, leading to NICD release (trans-activation). Binding of Notch and Delta molecules within the same cells occur with a rate constant k_c_ and typically leads to degradation of the ligand-receptor complex without further downstream effects (cis-inhibition). Notch, Delta, and NICD also degrade with basal rate constants (γ and γ_I_) due to single molecule degradation and dilution. While our model does not explicitly incorporate intracellular diffusion such as NICD translocation to the cell nucleus and Notch/Delta transport to the cell membrane, the activation (k_T_) and production (N_0_,D_0_) parameters implicitly consider the delay introduced by these processes.

Here, we study the effect of two types of noise: white and shot. The rightmost terms in Eq (1–2) consist of a noise amplitude multiplied by a Gaussian Normal distribution. To probe the effect of signaling noise on lateral inhibition, we only include stochasticity on the Notch and Delta rate equations. White noise intensity is independent of Notch and Delta copy numbers. The stochastic terms for Eq (1–2) are given by,

$$G_{N}(\sigma_{white})dW(t)=\sigma_{white}Normal(0,1)dt$$ (5)

$$G_{D}(\sigma_{white})dW(t)=\sigma_{white}Normal(0,1)dt$$ (6)

Conversely, shot noise intensity is proportional to the copy numbers of Notch and Delta. The functional forms of shot noise for Eq (1–2) are

$$G_{N}(\sigma_{shot})dW(t)=\sigma_{shot}\sqrt{Ndt}\;Normal(0,1)$$ (7)

$$G_{D}(\sigma_{shot})dW(t)=\sigma_{shot}\sqrt{Ddt}\;Normal(0,1)$$ (8)

In the absence of noise (i.e., the noise amplitudes in Eq (1) and (2) are set to zero), the single cell behaves either as a monostable or bistable switch depending on the levels of D_EXT_ and N_EXT_. The two monostable regions correspond to a Receiver state (high Notch, low Delta) and a Sender state (low Notch, high Delta). Inside the bistable region, the cell either falls into the Receiver or Sender states based on initial conditions with variable probability depending on the different (D_EXT_, N_EXT_) parameter combinations (Fig 1B).

The addition of noise introduces stochastic fluctuations around the stable fixed points of the deterministic model, which can be visualized in a pseudopotential landscape U(N,D) = -log(P(N,D)), where P(N,D) is the probability to observe levels of Notch and Delta equal to N and D, respectively, at any timestep once the simulation has equilibrated. Within the bistable parameter region, noise generates a landscape with two attractors, from which the location of the minima and the barrier height separating them can be quantified (Fig 1C). As expected, increasing either white or shot noise progressively decreases the height of the barrier separating Sender and Receiver states while increasing their basin of attraction (Figs 1D and S1A and S1B). Increasing white noise, however, progressively brings the two minima of the landscape closer. Conversely, the separation between Sender and Receiver states is maintained, if not increased, when increasing shot noise (S1C Fig). Therefore, even though the overall stability of these states decreases for larger noise amplitudes regardless of noise type, the Notch-Delta switch maintains a clear separation between states when exposed to shot noise. Conversely, strong white noise cannot be sustained effectively, leading to a progressive merging of the two states, in good agreement with previous studies of other small gene circuits [37].

The response to increasing noise levels underscores the necessity to quantify the intensity of these fluctuations based on biological parameters. A first baseline is the intrinsic noise arising from stochasticity of the chemical equations, which can be quantified with Gillespie-style simulations (S2 Fig, details of simulation in S1B Text). Interestingly, this intrinsic noise causes fluctuations of around 10 molecules in the copy numbers of Notch and Delta (Fig 1E, purple bars), much smaller than the typical values of all chemical rates in the system (order of 10^2^, Fig 1E, orange bars), and typical copy numbers of Notch, Delta, and NICD (order of 10^2^−10^3^, Fig 1E, yellow bars). Conversely, comparing the typical fluctuations of cellular Notch and Delta from the dynamics at different levels of shot noise against rate terms and copy number highlights different noise ranges (Fig 1E). Therefore, three qualitative ranges of noise can be defined–low, intermediate, and high–based on fluctuations comparable to intrinsic noise (order 10), chemical rates (order 10^2^), and typical copy numbers (10^3^) This trend is robustly observed in the case of white noise as well (S3 Fig).

## Modeling the multi-cell Notch-Delta landscape

### 1. A deterministic Notch-Delta multicell model leads to patterning disorder

The single cell model provided insight on the robustness of Sender and Receiver states in the presence of a noisy environment. In this case, the cell fate is governed by the exogenous ligands and receptors as well as noise. To understand the effect of noise on pattern formation, we generalize the lateral inhibition mechanism to a two-dimensional multicell scenario. By extending to a multicell scenario, the local behavior is now controlled by the binding of exogenous ligand and receptors to cellular ligand and receptors of neighbors (details of single cell model in the S1C Text). This allows us to analyze how the interactions of neighboring cells cause collective behaviors that lead to emergence of patterns.

While modeling cell-environment interactions provides clues on cell fate at the scale of single cells, the global properties of the multicellular system play an important role in the patterning. For instance, the exact pattern depends on the actual lattice shape, but lateral inhibition favors opposite cell fates leading to a semblance of order in the lattice structure. In the simplest case, a two-dimensional square lattice, the lateral inhibition mechanism leads to a stable checkerboard pattern with alternating Senders and Receivers [4,15]. We use this model to understand pattern formation from a nucleating event, such as in the fruit fly wing resulting in a checkerboard pattern, and pattern formation in a noisy environment, such as seen in diseased tissues.

**Fig 2:**
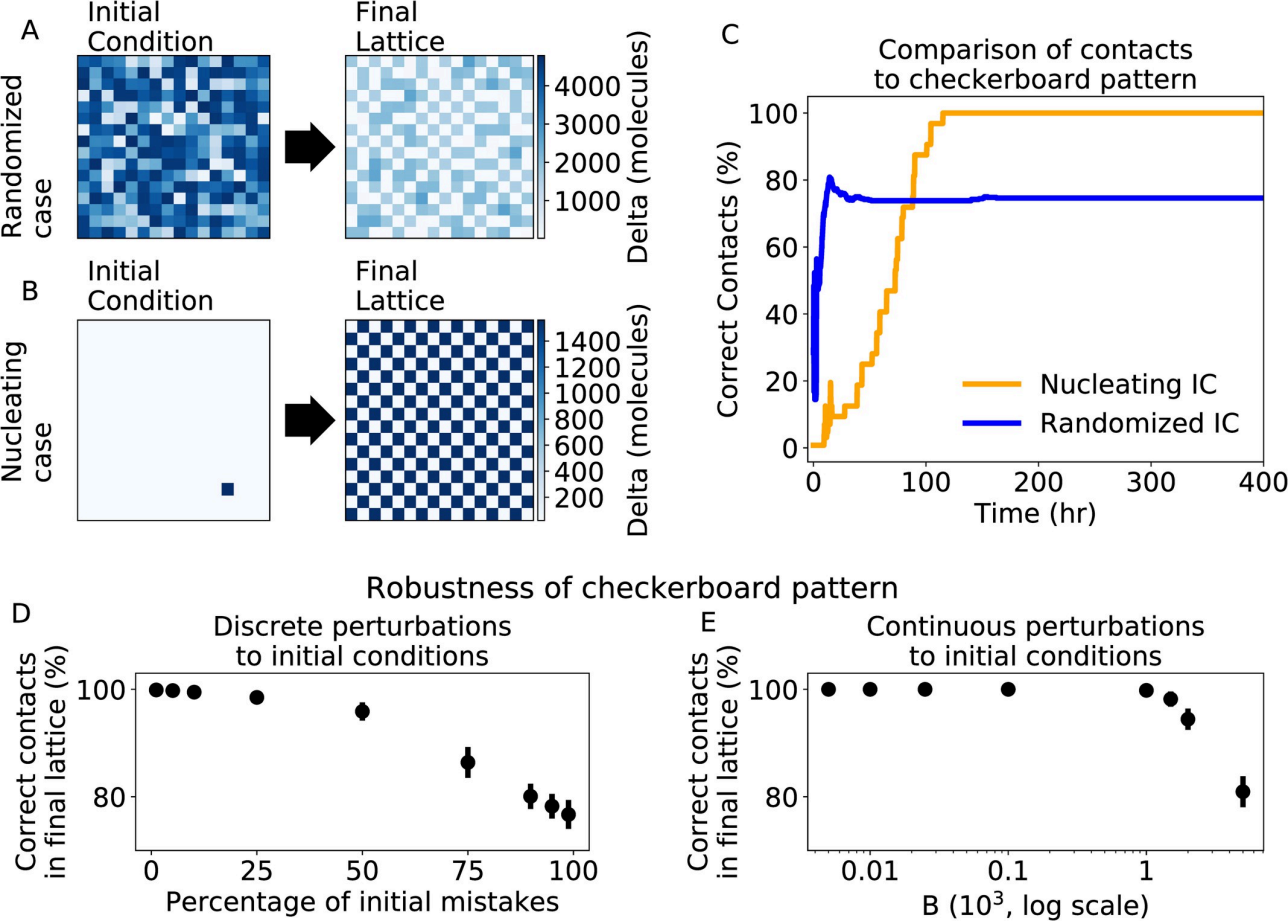
Patterning disorder in the noise-free multicell model. **(a)** the initial (left) and final (right) patterns starting from randomized initial conditions. The blue heatmap quantifies the cellular levels of Delta. **(b)** Same as (a) for a “nucleating” initial condition. **(c)** The percent of correct contacts as a function of time for the randomized initial conditions (blue) and the nucleating initial condition (orange) corresponding to panels (a) and(b). The lattice with randomized initial condition leads to a frustrated pattern. **(d)** The average percent of correct contacts as a function of percentage of mistakes in the initial checkerboard pattern. **(e)** The average percent of correct contacts as a function of B, the amplitude of the lattice perturbation normalized by 10^3^ (the magnitude of Notch and Delta copy numbers). For panels (d)-(e), percentages of correct contacts are calculated upon full equilibration and averaged over 20 independent simulations. The checkerboard pattern is robust for up to a quarter of mistakes in the lattice or a perturbation of magnitude 10^3^ molecules, comparable to the Notch and Delta copy numbers.

Before studying the temporal and spatial dynamics of pattern formation in a noisy environment, we first establish the deterministic properties of the model. In the absence of noise (σ = 0), a cell layer starting from randomized initial levels of Notch, Delta, and NICD relaxes to a frustrated pattern with many “incorrect” contacts (Sender/Sender and Receiver/Receiver) (Fig 2A and S1 Movie). Interestingly, an initial pattern with only one Sender surrounded by Receivers (or “nucleating” case) causes a spatio-temporal cascade that results in a perfect checkerboard pattern (Fig 2B and S2 Movie). Thus suggesting, in deterministic cases, only certain initial conditions allow the system to evolve to a checkerboard pattern.

To quantify the deviation from checkerboard patterning, we calculate the percent of “correct” (Sender/Receiver) contacts. Thus, 100% of correct contacts represents a perfect checkerboard pattern, a random pattern would have about 50% of correct contacts, and a pattern where all cells have the same fate would have no correct contacts. Analyzing the dynamics of the “nucleating” initial condition shows the system consistently increases in order and reaches a perfect checkerboard pattern on a timescale of about one hundred hours, qualitatively consistent with Notch-driven multicell patterning in multiple developmental systems [8]. Conversely, the randomized initial condition leads to a frustrated pattern with only ~75% correct contacts (Fig 2C).

To test how “incorrect” contacts (Sender/Sender or Receiver/Receiver) emerge during pattern formation, we set up several cell layers with very specific initial conditions. These initial conditions include (1) one quadrant of Senders with the rest Receivers, (2) one quadrant of Receivers with the rest Senders, (3) one row of Receivers with the rest Senders, (4) one row of Senders with rest the Receivers, and (5) the top half Receivers and the bottom half Senders (S4 Fig). None of the lattices reached a checkerboard pattern upon equilibration, indicating that the multicell Notch-Delta switch can easily remain “stuck” in configurations with “incorrect” contacts between Senders and Receivers, reminiscent of patterning mistakes in spin systems.

We further systematically analyzed the stability of the checkerboard pattern in the noise-free limit, by studying its response to different perturbations. First, discrete perturbations in a checkerboard pattern were created by randomly selecting a fixed fraction of cells in a checkerboard pattern and altering their cellular levels of Notch, Delta and NICD, resulting in an initial condition with a percentage of mistakes (i.e., artificially introducing defects into the checkerboard by changing the cell state from the “correct”). The checkerboard pattern can be recovered when less than a quarter of cells initially occupy an “incorrect” state. A higher percentage of mistakes, however, results in a disordered final pattern that deviates further from the checkerboard pattern as more mistakes are introduced in the initial lattice (Figs 2D and S5).

Furthermore, we tested a continuous perturbation by adding Gaussian noise to a perfect checkerboard initial condition. Specifically, each cell in a checkerboard lattice has a Gaussian random variable with mean, μ = 0, and standard deviation, B, added to the cellular values of Notch, Delta, and NICD. The final patterns exhibit disorder once the amplitude of the perturbation becomes comparable to the magnitude of Notch and Delta copy numbers (B = 1000), (Figs 2E and S6).

Thus, in the noise-free limit, the spatial cell distribution must either be initially very similar to the target checkboard pattern or exhibit a very specific initial pattern (e.g., nucleating) to be able to recover the checkerboard, while larger deviations lead to frustrated patterns. In analogy to the navigation of spin glass energy landscapes, there seem to be a multitude of basins representing both ordered (checkerboard) and disordered/frustrated patterns. Further, the robustness of the checkerboard pattern to small changes suggests the checkerboard basin is surrounded by basins representing increasingly disordered systems as the distance from the checkerboard basin increases.

These results are robust to local parameter variation, as seen by comparing the change in average NICD level for all cells in the simulation between the original model and model with a single altered parameter, (S7 Fig). Further, these results are also robust with respect to lattice size. A lattice size of 16x16 cells was originally chosen to ensure the convergence of the global properties, and specifically the percent of correct (Sender/Receiver) contacts. While the individual cell dynamics are expected to be consistent for any lattice size, small-size effect can arise for smaller lattices. Our analysis shows that the global properties for the chosen lattice size converge to values achieved by larger lattices (S8 Fig).

### 2. Optimal noise maximizes lateral inhibition ordering

To elucidate the role of noise in Notch-driven spatial patterning, we generalized the multicell model to include the effect of white and shot noise. In analogy to the single cell model, we generalize the definition of the pseudopotential landscape U = -log_10_P_multi_(N,D) to characterize Sender and Receiver cells (*Methods* section 1). Therefore, P_multi_(N,D) is the probability that any cell in the lattice will have a level of Notch and Delta corresponding to N and D as the simulation progresses. We consider a cell to be in the Sender attraction basin when its pseudopotential energy does not exceed a fixed threshold, chosen to be at a 10-fold difference to the Sender minimum on the pseudopotential landscape (S9 Fig). Likewise, for the Receiver, the cell must be within the threshold of the Receiver attraction basin.

**Fig 3:**
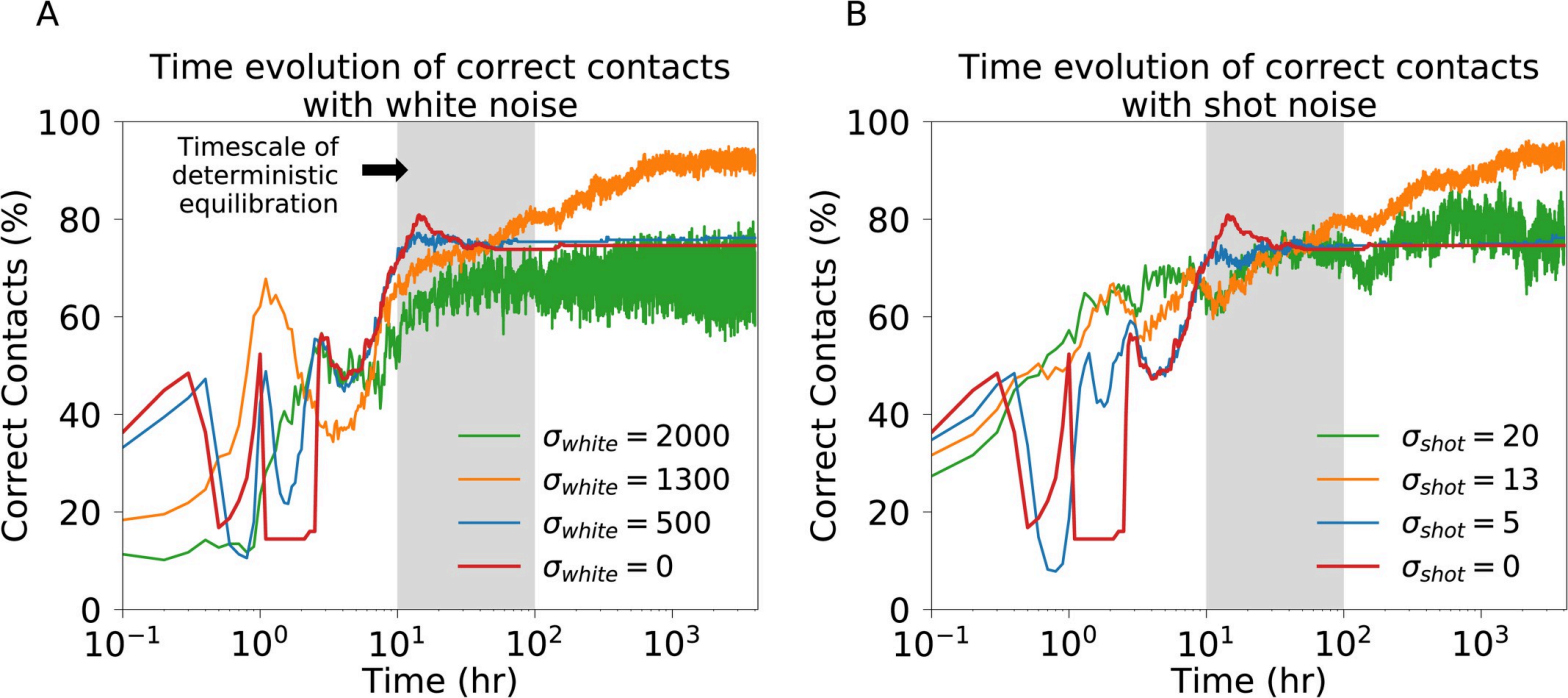
Time evolution of pattern formation in the multicellular system. **(a)** The percent of correct contacts over time for individual simulations with white noise amplitudes (σ_white_ = 0, 500, 1300, and 2000). The gray region shows the typical timescale of Notch equilibration and cell cycle. The systems start from randomized initial conditions and relax towards more ordered patterns within the first 10−10^2^ hr (i.e., Notch equilibration). Afterwards, the stochastic fluctuations dominate, leading to a separation in the order of systems based on noise amplitude. **(b)** Same as (a) but for corresponding shot noise levels (σ_shot_ = 0, 5, 13, and 20).

First, we study the dynamics of the correct contacts fraction in the initially randomized lattice as the amplitude of stochastic fluctuations increase. Near the start of the simulation ([1–10] hr), the system undergoes many drastic changes and quickly relaxes towards a more ordered pattern with around 60%-70% of correct contacts (Fig 3A for white noise and Fig 3B for shot noise). This is followed by a slower and jumpier equilibration process that separates the systems into distinct levels of correct contacts based on noise amplitude–zero, low, intermediate, or high noise–which correspond to fluctuations comparable to intrinsic noise (order 10 molecules), chemical rates (order 10^2^ molecules), and typical copy numbers (10^3^ molecules). Notably, the patterning order in the low and high noise regimes is similar to the deterministic model, whereas intermediate noise leads to a larger correct contact fraction (Fig 3A and 3B). Thus, the many jumps for the first few simulation hours followed by fluctuations around a single value suggests two timescales for final patterning. During the first stage, the system quickly relaxes towards a roughly ordered pattern on a timescale associated with Notch signaling equilibration and cell cycle which typically lie within the [10–100] hr interval. The second stage is dominated by fluctuations that modulate the pattern on a longer timescale ([100–1000] hr). The effect of stochastic fluctuations can be further decoupled from the chemical kinetics with simulations starting from a perfect checkerboard pattern. In this case, noise is the only source for disruption of the pattern; therefore, the fraction of correct contacts progressively separate based on noise level (S10 Fig).

A potential drawback in quantifying disorder based on Receiver-Sender contacts is the ambiguity in defining these cell states in the presence of high noise, as cellular Delta levels do not clearly separate between low (i.e., Receiver) and high (i.e., Sender) (S11A–S11C Fig). For this reason, we further define a ‘similarity’ patterning metric based solely on correlations of Delta levels between neighbors. When applied to the deterministic multicell trajectories of Fig 2C, the similarity yields analogous results to the correct contacts, thus showing the robustness of our analysis (S11D Fig). Comparing similarities at different noise amplitudes confirms the progressive separation of ordered and disordered systems based on noise amplitude, as the level of Delta is modulated even as the pattern is unchanged, allowing for a more apparent separation of order (S12 Fig). Additionally, in agreement with both the correct contact fraction and similarity, the time averaged correlation between the final lattice and lattices throughout the simulation shows the two distinct timescales corresponding with the fast chemical kinetics of the system and the subsequent relaxation guided by the stochastic fluctuations, demonstrating that noise operates after the chemical relaxation is achieved (S13 Fig).

While individual simulations provide insight into the timescales of relaxation, we look at the statistics of aggregated simulations to broadly understand pattern formation. Similar to the single cell system, the basins of attraction are modulated by the noise amplitude. When increasing the amplitude of shot noise, a clear separation is maintained between the Sender and Receiver minima (4A-C). Conversely, the states begin to slowly merge when increasing white noise (S9 Fig), thus confirming our model has consistent response to noise regardless of system size.

**Fig 4:**
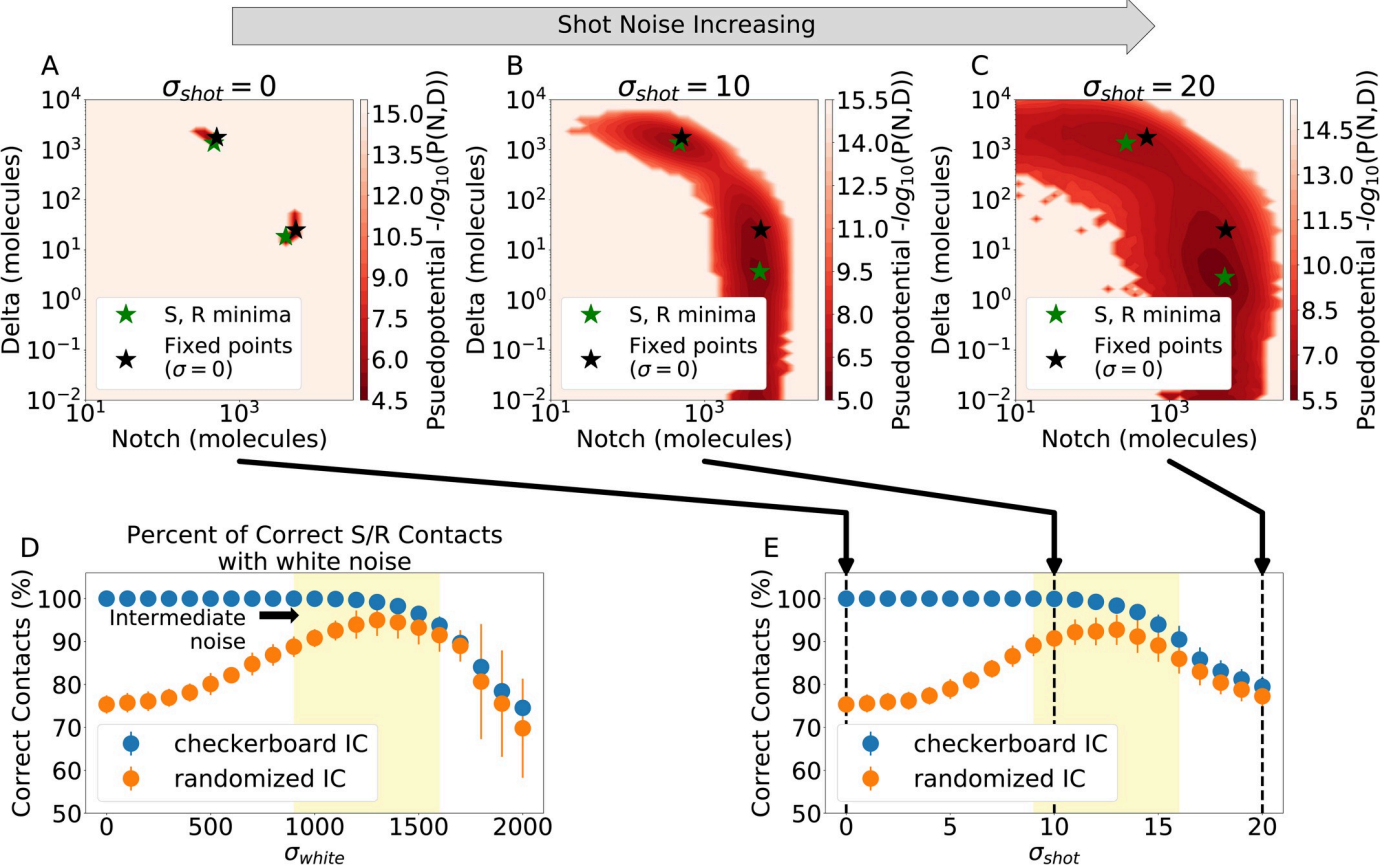
Stochastic influence on lateral inhibition and multicell pattern formation. **(a)** Pseudopotential landscape for the multicell model in the absence of noise (σ_shot_ = 0) for a single independent simulation. X- and y-coordinates represent cellular levels of Notch and Delta, respectively. Green starred dots depict the location of the landscape minima corresponding to S and R states, while black starred dots depict the location of the S and R states in a perfect checkerboard pattern. **(b)** Same as (a) but for σ_shot_ = 10. **(c)** Same as (a) but for σ_shot_ = 20. Panels (a)-(c) show that shot noise maintains separation of the Sender and Receiver states even as the system explores the state space. **(d)** The percentage of correct contacts (i.e., Sender-Receiver) as a function of white noise for random initial conditions (orange). **(e)** Same as (d) but for correct contacts as a function of shot noise. The lattice is influenced towards a more ordered system for intermediate levels of noise. For panels (d) and(e), results are averaged over 20 independent simulations of 9000 hr using random initial conditions after a 1000 hr relaxation period. For initially checkerboard systems, 20 independent simulations of 4000 hr are averaged after allowing for 1000 hr of relaxation.

We further analyzed the aggregated data by comparing the statistics of correct contacts at steady state when starting from either randomized or checkerboard initial conditions. This analysis highlights distinct responses in the three noise regimes (low, intermediate, and high). In the low noise regime, the randomized lattice exhibits disordered patterns qualitatively similar to the zero-noise limit case (~25% of incorrect contacts), whereas the checkerboard system maintains the checkerboard pattern with nearly 100% of correct contacts (left regions of Fig 4D and 4E). Therefore, low noise levels facilitate sparse exploration of the patterning landscape. In the intermediate noise regime (approximately 900<σ_white_<1600 and 9<σ_shot_<16), the patterning order of the randomized lattices increases and peaks around 92% and 95% of correct contacts (for σ_white_ = 13 and σ_white_ = 1300, respectively) before becoming more disordered in the high noise region (middle and left regions of Fig 4D and 4E). Further, the difference between the peak in correct contacts for systems starting from randomized initial conditions (i.e., the most ordered system for the shot and white noise cases) is equivalent when also accounting for the standard deviation across all simulations. Conversely, the initially checkerboard pattern becomes less ordered and progressively similar to the initially randomized system as noise increases (middle regions of Fig 4D and 4E). This peak in order implies that, at intermediate noise levels, the system can escape the local, frustrated minimum, explore the landscape and find ordered patterns that are nearly checkerboard. Finally, in the high noise regime, both randomized and checkerboard initial conditions progressively decrease their order as noise increases, suggesting the systems switch too quickly between different basins to relax into an ordered pattern (right regions of Fig 4D and 4E). Consistently, the most ordered patterns occur in the intermediate regime when inspecting the similarity patterning metric (S14 Fig), while confirming a proper one-to-one ratio of Sender and Receiver cells (S15 and S16 Figs), upon perturbations in the lattice initial condition (S17 and S18 Figs), and when varying lattice size (S19 Fig).

The beneficial effect of intermediate noise on order is further demonstrated by the distribution of correct contacts explored during the simulation (S21 Fig). There is little exploration of the landscape at low noise levels, while intermediate noise levels allow exploration of many configurations with high correct contact fractions, further supporting the analogy that the checkerboard pattern resides in the lowest energy minima and is surrounded by many shallow minima. Conversely, at high noise levels the lattice explores a wide range of configurations but is not able to attain ordered patterns.

Additionally, we determined that altering the way noise is implemented in the system can modify ordering. Treating the noise amplitudes of Notch and Delta as independent variables suggests that fluctuations on Delta have a slightly stronger effect in increasing ordering (S20 Fig). In other words, the pathway seems more sensitive to perturbations to levels of Delta ligands than Notch receptors.

Finally, we test the patterning response when including noise on NICD, which might correspond to fluctuations in NICD cleavage, transport and downstream gene transcription. Notably, the response of patterning order to increasing NICD noise mirrors the response observed for Notch and Delta noise, but at lower noise amplitude to account for smaller NICD copy number, both in the case of white and shot noise (S22 Fig). Taken together, the consistent responses to different noise amplitudes on Notch, Delta and NICD suggest a general relation between intermediate fluctuation levels and optimal patterning.

### 3. Intermediate noise maximizes order by selectively flipping incorrect cell states

To elucidate how patterning and error correction operate at different noise levels, we studied more systematically the statistics of cell switching and its dependence on the local cell environment. To define single cell transitions in a noisy multicell system, we take advantage of the pseudopotential landscape. To complete a transition, a cell must not only exit the attraction basin of its current state, but also enter the attraction basin of the opposite state. Following a parallel with the single cell model, a Receiver cell surrounded by a “Sender-like” environment should have a low switching rate, whereas a Receiver cell surrounded by a “Receiver-like” environment should have a higher switching rate.

**Fig 5:**
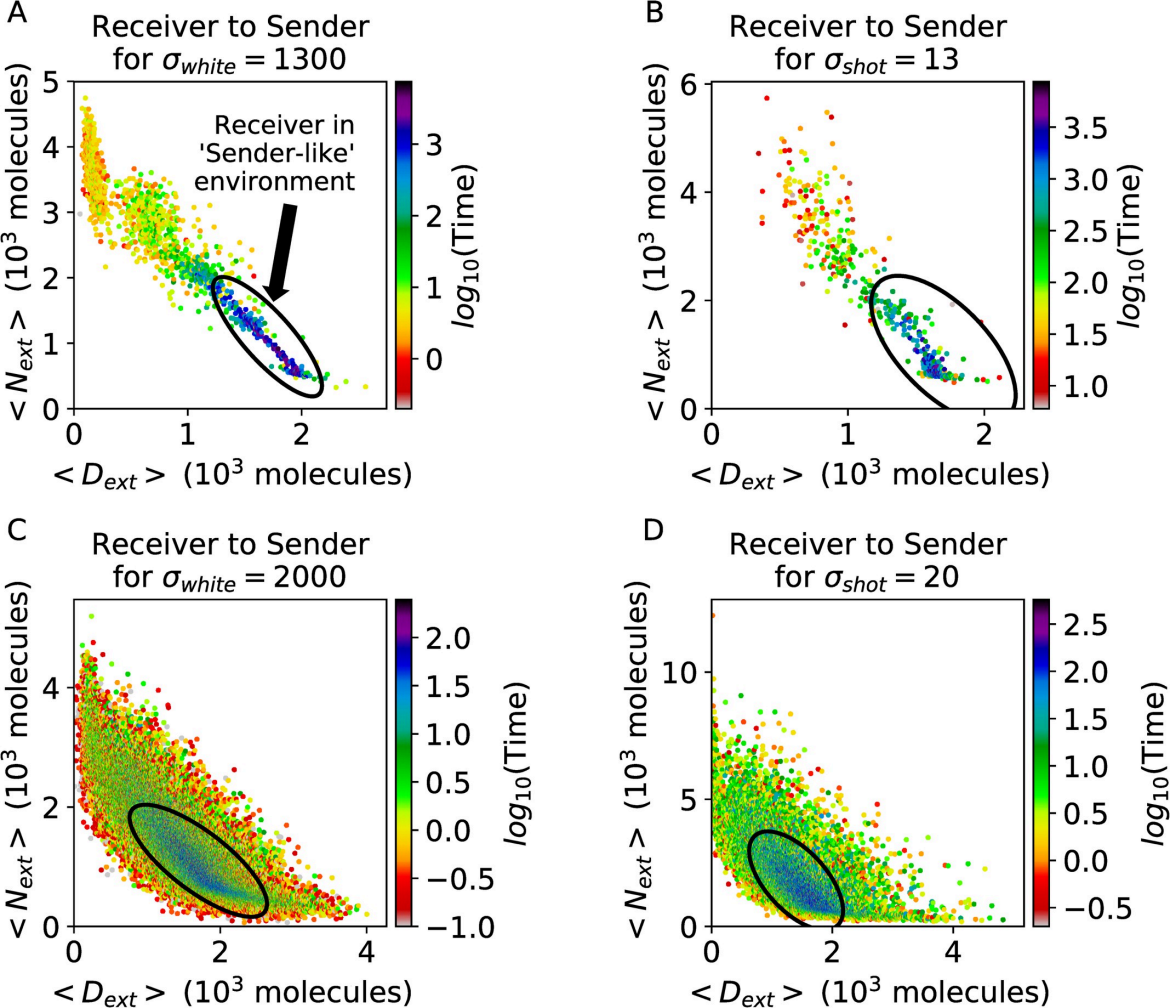
Timescales for single cell and lattice transitions. **(a)** The distribution of transition waiting times from Receiver to Sender as a function of N_EXT_ and D_EXT_ in a simulation starting from randomized initial conditions with white noise amplitude of σ_white_ = 1300 for 9000 hr after a 1000 hr relaxation period. Each dot represents the average switching event, while the x- and y-coordinates represent the average levels of Delta and Notch in neighboring cells during the transition. The color scale depicts the waiting time before the transition event. The “correct” state region (a Receiver surrounded by Sender-like environment) is circled, and dots within this region exhibit the longest transition waiting time. **(b)** Same as (a) but for σ_shot_ = 13. **(c)** Same as (a) but at a high white noise level (σ_white_ = 2000). These larger fluctuations also flip cells from the “correct” to “incorrect” state. **(d)** Same as (a) but for high shot noise (σ_shot_ = 20).

To quantify the switching as a function of the cell’s local environment, we estimate the transition waiting times between Sender and Receiver states as a function of external Notch and Delta defined as the average over the cell’s nearest neighbors. In other words, the transition waiting time represents the time spent in a state (Sender or Receiver) before switching to the opposite state. At intermediate noise levels, the Receiver state is stable (10^3^−10^4^ hr) when surrounded by a “Sender-like” (high Delta, low Notch) environment, while switching occurs on much shorter timescales (1–10 hr, comparable or shorter to the timescale of Notch equilibration) when surrounded by a “Receiver-like” (low Delta, high Notch) environment (Fig 5A and 5B). Consistently, the Sender state follows the opposite trend, being stable when the environment behaves as a Receiver and unstable when the environment behaves as a Sender (S23 Fig). The stability of cells in the “correct” environment suggests highly ordered patterns lie within deep minima on the landscape while disordered patterns are represented by shallow minima. Strikingly, the connection between external signal (Sender-like vs Receiver-like environment) and transition time is weaker for cases of very high noise (σ_white_ = 2000, σ_shot_ = 20) due to the destabilization of the checkerboard pattern. At these high noise amplitudes, fast transitions were observed when cells occupied the “correct” and “incorrect” states, suggesting stochastic fluctuations are larger than the energy barriers separating ordered and frustrated configurations (Fig 5C and 5D). Therefore, intermediate noise levels provide enough perturbation to “flip” cells that occupy an incorrect state, whereas a very strong noise also enforce transitions in cells that occupy the correct state, thus decreasing the overall ordering. This trend is consistently observed when comparing transition times as a function of Sender/Receiver neighbors instead of average external Delta/Notch. The greatest stability exists for cells in the “correct” environment (S24 and S25 Figs).

## Discussion

Notch-Delta signaling plays a ubiquitous role in multicellular pattern formation in different physiological and pathological contexts by promoting lateral inhibition between Sender cells (low Notch, high Delta) and Receiver cells (high Notch, low Delta) [10,38]. While noise plays a key role in this signaling [20–23], most of the existing theoretical analysis focuses on deterministic models [12,13,15–17]. Here, we developed a stochastic model of Notch-Delta biochemical signaling to elucidate the role of fluctuations during Notch-driven spatial patterning formation. Our results suggest that intermediate levels of stochastic fluctuations promote an ordered patterning by proofreading the pattern and forcing cells to “flip” to their “correct” state. The fluctuations required for ordered patterning are greater than the intrinsic noise arising from Gillespie-style simulations, but substantially smaller than the typical copy numbers of Notch and Delta.

The connection between multicell patterning and noise was investigated using a two-dimensional model of cells arranged on a square lattice. In the absence of noise, a checkerboard pattern steady state with alternating Sender and Receiver cells could be recovered when the initial pattern was close to the final checkerboard pattern or in the presence of other, specific initial conditions, such as the ‘seeding’ of a single Sender cell. Randomized initial conditions, however, led to disordered steady states, suggesting the existence of a complex landscape with several local attractors. In the presence of weak stochastic fluctuations, the lattice stays near the comparable zero-noise disordered state; conversely, in presence of high noise the system cannot stabilize the ordered pattern and explores disordered patterns in the configurational landscape. Strikingly, intermediate noise levels lead to an optimal patterning order. Specifically, analysis of single cell transition trajectories within the lattice demonstrate that intermediate noise can serve as a mechanism for error correction by “flipping” cells to the “correct” state. Our findings that the Notch multicell system can be trapped in a frustrated state if not initially seeded for checkerboard (in the deterministic case) or without the proper level of stochastic fluctuations, agrees with previous results which suggest stochastic fluctuations are critical to correct patterning [20–22,32]. Our study focused on two types of noise, white and shot, which have been previously shown to capture different regimes of stochastic fluctuations in gene regulatory networks [29]. Strikingly, the pattern is highly ordered in the intermediate noise case for both white and shot noise, suggesting noise type does not have a major influence on the global properties. Further, the pattern is more ordered when the fluctuations in cellular Delta are greater than cellular Notch. The higher susceptibility to noise is at least partially dependent on the lower copy number of Delta in the model. Thus, it might be interesting in future investigations to quantitatively gauge copy numbers experimentally to test this prediction. Our results, which focus specifically on the Notch circuit, agree well with more coarse-grained models of lateral inhibition patterning based on Landau model [28] and cellular automata model [33], thus confirming this is a biological phenomenon critical to Notch-Delta mediated pattern formation.

Methods from statistical mechanics, and the Ising model in particular, have been previously used to understand a multitude of biological problems including neural networks, bird flocking, and protein folding [39–42]. In analogy with the energy landscape of conventional spin models, the multi-stability of biochemical and gene regulatory networks can be described by a pseudopotential landscape [43]. This effective energy landscape approach has been previously applied to explain cell fate transitions in other biological systems, such as the Epithelial-Mesenchymal Transition (EMT) in cancer [44]. Qualitatively comparing the antiferromagnetic Ising model, and the associated energy landscape, to Notch-mediated pattern formation provides insights on how patterns achieve ordering. It has been shown for low temperature spin glasses, that the free energy landscape is very rugged and has many valleys in which the systems become trapped in frustrated states [45–46]. These barriers between valleys block the access of the system to the lowest energy state unless there is an external source modulating the free energy landscape [47].

Following the parallel with a spin glass landscape, our results can be interpreted as follows. In the deterministic case (i.e., zero-temperature limit), Notch-Delta systems can only reach their lowest energy state (i.e., checkerboard) if there is a downhill path they can traverse through the landscape, whereas systems with randomized initial conditions remain trapped in frustrated states. Stochastic fluctuations allow the multicell system to navigate the landscape, overcome energy barriers and access low-energy states. While low noise is sufficient to escape local, frustrated minima and reach slightly more ordered systems, intermediate noise enables a more thorough exploration of the landscape to reach very ordered, low energy states. Fluctuations in the high noise case, however, become larger than the energy barriers separating different stable patterning configurations, causing the system to remain unstable and switch between high and low energy states. Thus, the spin glass energy landscape can provide a useful framework to understand the system-wide effect of noise on Notch-mediated pattern formation.

Given that pattern formation in biological systems is not instantaneous, we considered how the temporal dynamics of individual cells contributed to pattern formation. Our results suggest a short timescale corresponding with the typical equilibration time of the Notch circuit (10−10^2^ hr) where the system attains a rough ordering with many mistakes, and a longer timescale where stochastic fluctuations further ‘proofread’ the pattern and allow the system to relax towards order. This timescale separation potentially helps explain different patterns observed experimentally. For example, sprouting angiogenesis integrates fate differentiation, proliferation, and migration on timescales comparable with the Notch circuit equilibration (i.e., tens of hours), thus potentially not providing endothelial cells with enough time to navigate the ‘patterning landscape.’ In this case, lateral inhibition between migrating Tip cells (i.e., Senders) and proliferating Stalk cells (i.e., Receivers) organize the formation of a new blood vessel from the existing lumen [48]. Interestingly, patterning disorder and uneven spacing between sprouts has been recently reported in Human umbilical vein endothelial cells (HUMEC) cultivated *in vitro* [49]. Similarly, the early developmental stages of the avian auditory organ (E8-E9) are characterized by frequent contacts between hair cells (i.e., Senders). This patterning is corrected at later stages, after approximately 4–5 more days, despite unchanged ratios of hair and supporting cells (i.e., Receivers), thus suggesting a role for stochastic fluctuations in cell rearrangement [50]. Therefore, it can be suggested that due to the predicted timescale separation between chemical equilibration and noise-driven patterning correction, stochastic fluctuations might not influence pattern formation on short molecular timescales leading to disordered patterns, but instead affect biological processes that takes place on longer time scales.

Here, we focused on a core Notch-Delta circuit that captures the general aspects of lateral inhibition while disregarding context-specific features which might be pivotal to understand the signaling in specific circumstances. This framework, however, could be generalized in the future to inject prior knowledge on a greater range of biological questions. For instance, epithelial cells often organize in nearly hexagonal geometries that exhibit a lowest energy state where Senders are surrounded by six Receivers [18], as seen for instance in the patterning of bristles during fruit fly development [38]. Also, the ‘standard’ Notch-Delta signaling can be modified by additional mechanisms such as long-range interactions via diffusible ligands [51], variable cell-cell contact area [52], and intracellular heterogeneity arising from spatial diffusion [49]. In particular, Koon and collaborators recently modeled intracellular Notch diffusion in the context of Tip-Stalk lateral inhibition and proposed intracellular heterogeneity as a possible mechanism to enable variable spacing between Tip cells [49]. This model primarily focused on one-dimensional cell chains, and therefore the implications of intracellular spatial dynamics in presence of more complex spatial constraints remain currently unexplored.

Moreover, the circuit topology and emergent properties of the Notch-Delta switch can be modified by additional molecular details. First, ligands of the Jagged family can compete with Delta and instead lead to lateral induction, or spatial propagation of a similar hybrid Sender/Receiver phenotype. Jagged plays a critical role in coordinating collective cell migration during cancer invasion [12,53]. It was previously shown for tristable cell switches that shot noise tends to destabilize intermediate cell states while white noise stabilizes them [29]. Therefore, it can be speculated that white and shot noise might have opposite effects on the hybrid Sender/Receiver state [29]. Additionally, the glycosyltransferase Fringe can modulate the binding rates of Delta and Jagged ligands, thus effectively enhancing Notch-Delta signaling and perhaps sustaining a more robust checkerboard pattern despite noise fluctuations [12,54–55]. Moreover, feedback regulation and time delay between Delta and HES/HEY leads to spatiotemporal oscillations during somitogenesis [5]. Generalizing the current modeling framework to include these additional regulations could in the future provide even more biological insight into the function of Notch.

Finally, Notch regulates, and is in turn sensitive to, mechanical cues in the cellular microenvironment, hence raising interesting questions about the integration of biochemical and mechanical regulation of Notch signaling [56]. Thus, it would be of interest to quantify how cell packing geometry and modifications of Notch signaling modulate the interplay between stochastic fluctuations and patterning. Confirming that optimal noise levels can influence these systems towards the lowest energy state would provide further evidence that noise is critical to Notch-driven patterning.

## Methods

### 1. Identification of Sender and Receiver states in the multicell model

Spatial constraints and noise give rise to a continuous spectrum of Notch and Delta levels in the multicell model, thus complicating the classification of cells as Sender or Receivers. To determine the state of individual cells and whether the pattern is exactly checkerboard or a checkerboard with a few mistakes, we define the Sender and Receiver states based on the pseudopotential

$$U(N,D)=-\log_{10}\;P(N,D)$$ (9)

where P(N, D) is the probability of a cell to have a level of N and D molecules of Notch and Delta, respectively. The probability distribution of the multicell layer, P_multi_(N,D), is calculated for each set of initial conditions by aggregating the level of Notch and Delta in all cells of the square lattice starting once the system has relaxed/equilibrated (relaxation time is defined as t = 1000 hr after the beginning of the simulation) until the simulation ends, with timestep dt = 0.1hr, to construct the landscape.

In the pseudopotential, the two deepest minima are calculated and labeled as either Sender (Delta_minimum_>Notch_minimum_) or Receiver(Delta_minimum_<Notch_minimum_). For the Sender, the attraction basin is defined as the region of the landscape where the value of P_multi_(N, D) is increased by at most a 10-fold difference from the value in the minimum (P_multi,Sender_). The attraction basin for the Receiver state is defined likewise. A cell that is initially classified as a Sender will switch to the Receiver state if and only if it crosses the threshold to enter the Receiver basin (vice versa for a cell initially classified as Receiver). This condition prevents false positive state switches when large fluctuations transiently displace cells outside of their state thresholds.

### 2. Quantification of pattern disorder

When analyzing the final equilibrated results of the model, we looked at individual cells and the entire lattice collectively. Given that the Sender and Receiver states have Delta values two orders of magnitude different, we developed the similarity metric (S) that defines the distance to checkerboard using only the value of Delta within the cells. The benefit is we do not need to go through the analysis of identifying which state the cell is in while determining how close the pattern is to checkerboard. Defining the similarity metric using

$$S=\frac{1}{4N_{rows}}\sum_{i=1}^{N_{rows}}\left(1-r(x_{i},x_{i+1})\right)+\frac{1}{4N_{cols}}\sum_{j=1}^{N_{cols}}\left(1-r(x_{j},x_{j+1})\right),$$ (10)

where

$$r(x,y)=\frac{\sum_{k}\left(x_{k}-\bar{x})(y_{k}-\bar{y}\right)}{\sigma_{x}\sigma_{y}},$$ (11)

allows us to handle the continuous variables and shifting of the Sender/Receiver states. While these definitions work for most cases, if either the row or column has the exact same value of Delta then the similarity metric will not provide an accurate portrayal of the pattern, thus it is important to analyze the final patterns and ensure the similarity is near the expected value (e.g., near S = 1 if the pattern looks checkerboard).

We also compute the number of correct contacts in the lattice where a correct contact is defined as two adjacent cells that have opposite fates (S/R or R/S). Each cell in the square lattice can have up to 4 correct contacts with the total number of correct contacts in the lattice maximally 2(NxN) for a square lattice of length N cells for N> = 3. The number of correct contacts for each cell is defined as

$$CC_{i}=\frac{1}{2}\sum_{nn}\delta (state_{i},\;Sender)\delta (state_{nn},\;Receiver)+\delta (state_{i},\;Receiver)\delta (state_{nn},\;Sender),$$ (12)

Where the state is determined based on the pseudopotential landscape. If the cell is within the thresholds of the Notch and Delta values of the Sender basin then it is labeled Sender, and similarly for the Receiver state (details in previous section).

### 3. Lattice time correlation

The Receiver and Sender states of the Notch-Delta pathway can be transformed to the Up and Down states of the Ising model with a transformation where R = >1 and S = >-1. This transformation is defined using

$$s_{i}=\left\{ \begin{array}{c} 1,N_{i}>D_{i}\\ -1,D_{i}>N_{i} \end{array}\right.$$ (13)

where the continuous (N, D) variable system is transformed to a ±1 discrete spin system.

Also, using these transformed states, we can compute the time averaged correlation (q) [57] of each lattice with the initial or final lattice. These two equations can be used to quantify the timescale of similarity between the initial pattern and pattern at a later time

$$q_{initial}(t)=\frac{1}{N}\sum_{i}^{N}{\langle s_{i}(t=0)s_{i}(t)\rangle }_{T},$$ (14)

and between the final pattern and a pattern at an earlier time

$$q_{final}(t)=\frac{1}{N}\sum_{i}^{N}{\langle s_{i}(t=t_{f})s_{i}(t)\rangle }_{T}.$$ (15)

### 4. Calculation of transition waiting times

When calculating the transition time, we use the Sender/Receiver definitions mentioned previously. A successful transition occurs when a cell leaves its current attraction basin (Receive or Sender) and enters the opposite basin of attraction (Sender or Receiver). The transition waiting time begins after a successful transition (Receiver to Sender or Sender to Receiver). It then ends once the reverse transition is successful (Sender to Receiver or Receiver to Sender). The transition waiting time includes both the time spent in a basin of attraction (Sender or Receiver) and the time transitioning between basins (Sender to Receiver or vice versa). This accounts for fluctuations around the thresholds for the attraction basins and reduces the likelihood of misclassifying short or long transition times. Since the cell transitions are tied to the value of Notch and Delta in the neighboring cells (N_EXT_ and D_EXT_), we calculate the transition times at values of N_EXT_ and D_EXT_. To better identify the transitions, and since Notch and Delta are continuous variables, we bin the data by the ranges N = [0,8000] with a step of 10 and D = [0,6000] with a step of 1 and compute the average time for each pair of N and D. Likewise, we can also calculate the transition times as a function of neighbors that are Senders and Receivers.

## Supporting information

S1 Text Simulation Details. A. Details of model and simulation for Notch-Delta switch. B. Details of the one cell Gillespie model. C. Simulation details of multicell model.
(PDF)

S1 Movie Pattern formation starting from randomized initial conditions. The similarity (left) propagating as a function of time at the same speed as the pattern forms in the lattice (right) for a randomized initial lattice over the first 300 hr of the simulation for the deterministic case.
(MP4)

S2 Movie Pattern formation starting from the nucleating case. The similarity (left) propagating at the same time as the pattern forms in the lattice (right) for the nucleating case over the first 300 hr of the deterministic case ending in the checkerboard pattern.
(MP4)

S1 Fig The effect of noise in a single cell system on the pseudopotential and the probability of a state to be S or R. **(a)** Difference between barrier height from Sender to Receiver state (Δ_S_) and barrier height from Receiver to Sender state (Δ_R_) as a function of external Delta ligands (D_EXT_, x-axis) and Notch receptors (N_EXT_, y-axis). Four panels show increasing levels of shot noise. **(b)** Same as (a) for varying levels of white noise. **(c)** Pseudopotential landscape for increasing levels of white noise (top) and shot noise (bottom). White starred dots highlight the location of the stable fixed points of the corresponding deterministic model, while green starred points show the location of the landscape minima. For panel (c), D_EXT_ and N_EXT_ have the same values as in *Fig 1*(b).
(TIF)

S2 Fig Intrinsic noise from one cell Notch-Delta Gillespie simulation. **(a)** The probability of a cell being a Receiver for a particular level of exogenous Delta and Notch. The results of ten simulations were averaged to develop this phase plane. **(b)** c standard deviation of Delta in a cell for all N_EXT_ and D_EXT_ pairs of (a) corresponding to the intrinsic noise of cellular Delta. **(c)** The same as (b) for cellular Notch.
(TIF)

S3 Fig The comparison of white noise to other parameters of the one-cell system. The purple bars are the intrinsic noise calculated from Gillespie simulations and have a magnitude of about 10. The orange bars are the chemical terms averaged over the Sender and Receiver values and have a magnitude of about 10^2^. The yellow bars are the average N, D, and I copy numbers with magnitude averaging 10^3^. The bars show approximately which levels of added noise in the system are comparable to various categories (intrinsic noise, chemical terms, or cellular concentrations).
(TIF)

S4 Fig Deterministic multicell simulation results for specific patterns made of Senders and Receivers only. The initial pattern (left), final pattern (middle left), similarity (middle right), and percent of correct contacts (right) as a function of time for a quadrant of Senders, a quadrant of Receivers, a line of Senders, a line of Receivers, and half Senders and half Receivers, (a)-(f) respectively.
(TIF)

S5 Fig Results of deterministic multicell simulations of patterns starting from lattices with discrete perturbations. Example of initial pattern (left), final pattern (middle left), similarity metric (middle right), and percent correct contacts (right) as a function of time for deterministic simulations for initial checkerboard lattices with discrete perturbations. The number of initial mistakes in the 256-cell lattice are 3 (1.2%), 26 (10.2%), 128 (50%), 192 (75%), 243 (94.9%), and 253 (98.8%) mistakes for (a)-(f), respectively.
(TIF)

S6 Fig Results of deterministic multicell simulations starting from lattices with continuous perturbations. Example of deterministic results for initial checkerboard lattices with continuous perturbations showing the initial pattern (left), final pattern (middle left), similarity metric (middle right), and percent of correct contacts (right) as a function of time. The initial conditions for the simulations had an added Gaussian random variable of mean μ = 0 and standard deviation of B = 1, 10, 25, 100, 1000, and 2000 (a-f, respectively).
(TIF)

S7 Fig Sensitivity analysis for the multicell model at different noise amplitudes. The difference in NICD when a single parameter is changed compared to model with no changed parameters. The results are the average for a multicell system once the system has equilibrated. **(a)** For deterministic model showing robustness. **(b)-(d)** Same as (a) but for the stochastic model with white noise at σ_white_ = 700, 1300, and 2000. **(e-g)** Same as (a) but for stochastic model where σ_shot_ = 7,13,20.
(TIF)

S8 Fig Comparison of pattern convergence between different lattice sizes at various levels of noise. **(a)** The correct contacts for deterministic multicell simulations with a square cell length of 4 (blue), 8 (orange), 16 (green), and 32 (red). The systems with lattice length larger than 4 converge to the same solution. **(b-d)** Same as (a) but for stochastic model where σ_white_ = 700, 1300, and 2000. **(e-g)** Same as (a) but for stochastic model where σ_shot_ = 7,13,20. These results show that lattices of size 16 and 32 have converged to the same result, therefore we can simply our calculations and use the smaller lattice of length 16 cells.
(TIF)

S9 Fig The multicell pseudopotentials when white noise is included in the system. The pseudopotential landscapes U = -log_10_ P(N,D), where P(N,D) is the probability of a cell having a level of Notch and Delta equal to N and D, respectively. The green stars are the location of the pseudopotential minima, while the black stars represent the location of the Sender and Receiver states in a perfect checkerboard pattern without noise. The dotted lines depict the thresholds for the Sender and Receiver states. If the value of Notch and Delta of the cell are within a 10-fold difference from the Notch and Delta of the closest minima, then the cell is considered as in that basin of attraction. (a)-(d) Pseudopotential landscapes with stochastic fluctuation amplitudes of σ_shot_ = 0, 7, 13, and 20. (e)-(f) Pseudopotential landscapes with σ_white_ = 0, 700, 1300, and 2000.
(TIF)

S10 Fig The percent of correct contacts as a function of time for an initially checkerboard system. (a) The correct contacts or deterministic case (red), σ_white_ = 50 (green), σ_white_ = 130 (orange), and σ_white_ = 200(blue). (b) The correct contacts for the deterministic case (red), σ_shot_ = 5 (green), σ_shot_ = 13 (orange), and σ_shot_ = 20 (blue).
(TIF)

S11 Fig The similarity metric is based on distribution of Delta for all cells in a lattice. (a) The probability density of Delta molecules in all cells after the system has relaxed starting from randomized initial conditions with no applied noise for a single independent simulation. This density shows two distinct cell populations, Sender on the right and Receiver on the left. (b) Same as (a) but for σ_white_ = 2000 showing the populations are no longer distinct. (c) Same as (a) but for σ_white_ = 20. For (a), the distribution is computed for a representative sample of the cells in the lattice (6.25%) for a simulation starting from randomized initial conditions after equilibration to steady state (1000 hr). For (b) and (c) the distributions are computed for the sample of cells after the system has relaxed for 1000 hr. (d) The similarity to checkerboard starting from randomized initial conditions (blue) and the nucleating initial condition (lattice with one Sender and all other Receivers). Both are deterministic simulations showing the first 400 hr during which the simulation equilibrates to its final pattern (frustrated or checkerboard, respectively).
(TIF)

S12 Fig Comparing the similarity as the pattern evolves at different levels of noise. (a) The similarity as a function of time for randomized initial conditions showing the correlation of Delta in the cells throughout the lattice for the deterministic case (red, *σ*_*white*_ = 0), low noise case (green, σ_white_ = 500), medium noise case (orange σ_white_ = 1300), and high noise (blue, σ_white_ = 2000). (b) Same as (a) but with corresponding levels of shot noise (σ_shot_ = 0, 5, 13, 20). (c) Same as (a) but for checkerboard initial conditions. (d) Same as (b) but for checkerboard initial conditions.
(TIF)

S13 Fig The time average autocorrelation of the patterns starting from randomized initial conditions. (a) The time averaged autocorrelation function (q) comparing lattices with the final lattice pattern of the system in a stochastic system with σ_white_ = 500 and σ_white_ = 1300. The pattern begins to converge toward the final lattice pattern at around 10 hr. (b) In a stochastic system with σ_shot_ = 5 and σ_shot_ = 13, the time averaged autocorrelation function comparing lattices to the final lattice. The lattices converge towards a more ordered system starting at about 10 hr. The gray area is the typical timescale for the Notch system to equilibrate. The blue curves (intermediate noise) show a large slope in the grey region corresponding to fast equilibration driven by chemical kinetics and a smaller slope on the rightmost region of the plots corresponding to the error-correction driven by stochastic fluctuations.
(TIF)

S14 Fig The similarity metric as a function of noise shows increased order in the intermediate noise regime. (a) The similarity metric for randomized initial conditions (orange) and checkerboard initial conditions (blue) as a function of increasing white noise amplitude. (b) The same as (a) for shot noise. For all simulations there were 1000 hr allowed for the system to relax. For the randomized initial conditions, the results were averaged over 9000 hr for 20 independent simulations. The initially checkerboard system was averaged over 400 hr for 20 distinct simulations. The trends seen in the similarity metric mimic those exhibited by the correct contacts as a function of white or shot noise. While the similarity metric drops below S = 1 for the checkerboard initial condition more quickly than the fraction of correct contacts changes (see Fig 4), the steady decrease in the similarity metric is representative of the modulation and a loss of separation in the Delta values of the Sender and Receiver cell (see again S7 Fig).
(TIF)

S15 Fig The percent of Receivers and Senders for randomized initial conditions. (a) The average percent of Receivers and Senders as a function of increasing white noise amplitude. (b) same as (a) but for shot noise. The simulations are for lattices with randomized initial conditions. The lattices were allowed to relax for 1000 hr and then the results were averaged over the last 9000 hr of the 20 independent simulations. Throughout the intermediate noise regime nearly half of the cells are in the Sender state and the other half are in the Receiver state, confirming systems in this regime has the correct ratio of Senders to Receivers to achieve a checkerboard patterning.
(TIF)

S16 Fig The percent of Receivers and Senders starting from checkerboard initial conditions. (a) The average percent of Receivers and Senders as a function of increasing white noise amplitude for initially checkerboard lattices. (b) same as (a) but for shot noise. The lattices were allowed to relax for 1000 hr and then the results were averaged over the last 4000 hr of 20 distinct simulations. Throughout the low and intermediate noise regime nearly half of the cells are in the Sender state and the other half are in the Receiver state, confirming the systems have the correct ratio of Senders to Receivers to achieve a checkerboard patterning.
(TIF)

S17 Fig The similarity metric and correct contacts as a function of noise starting from a checkerboard with discrete perturbations shows improved order in the intermediate noise regime. (a) The similarity as a function of increasing white noise amplitude for checkerboard lattices with discrete perturbations of 13, 64, and 230 mistakes (corresponding to 5%, 25%, and 90% of mistakes in the lattice). (b) same as a but for shot noise. (c) same as (a) but for correct contacts as white noise increases. (d) same as (c) but for shot noise. The simulations were allowed to relax for 1000 hr and then averaged over the last 4000 hr of 20 simulations with different initial conditions. The checkerboard lattices with discrete perturbations follow a trend analogous to the randomized initial lattice where the systems are most ordered in the intermediate regime.
(TIF)

S18 Fig The similarity metric and correct contacts for checkboard with continuous perturbation initial conditions. (a) The similarity metric with increasing stochastic fluctuations from white noise for a checkerboard initial condition with an added Gaussian random variable of mean μ = 0 and standard deviation of B = 50, 1500, and 5000. (b) same as (a) but for shot noise. (c) Same as (a) but for percent of correct contacts as a function of increasing white noise. (d) Same as (c) but for shot noise. The simulations were allowed to relax for 1000 hr and then averaged over the last 4000 hr of 20 simulations with different initial conditions. Interestingly, in the presence of stochastic fluctuations, checkerboard lattices with continuous perturbations to all cells behave comparably to the checkerboard initial condition when perturbations are small and correspond to the randomized initial conditions when perturbations are larger.
(TIF)

S19 Fig The similarity metric and correct contacts for square lattices of length 4, 8, and 32 cells. (a) The similarity metric as a function of white noise for square lattices of length 4, 8, and 32 (square lattice of length 16 is in the main text). (b) Same as (a) but for shot noise. (c) same as (a) but for correct contacts as a function of increasing white noise. (d) Same as (c) for shot noise. There were 20 simulations starting with different initial conditions and averaged over 4000 hr after they were allowed to relax for 1000 hr. The response to noise is consistently observed across lattice sizes, with the trend being more robust as the size of the lattice increases.
(TIF)

S20 Fig The percent of correct contacts in a multicell lattice as noise on Notch and Delta are varied independently. The correct contacts are an averaged after a system has relaxed for 1000 hr. **(a)** The percent of correct contacts as the level of white noise added to cellular Notch (y) and Delta (x) changes. A slope of one is consistent with results from the main text. The results are nearly symmetric with higher noise on Delta correlated with slightly more ordered patterns at high noise levels. **(b)** Same as (a) for shot noise. The results are not as symmetric in this case with higher levels of noise on Delta correlated with greater ordered compared to the same level of noise on Notch. The results are averaged over 20 independent simulations starting from different randomized initial conditions after letting the system relax for 1000hr.
(TIF)

S21 Fig The pseudopotential as a function of correct contacts. (a) The pseudopotential landscape U = -log_10_ P(σ,CC) as a function of correct contacts fraction and white noise amplitude (σ_white_). (b) Same as (a) but for shot noise amplitude (σ_shot_). The landscapes were constructed using data from 20 independent simulations.
(TIF)

S22 Fig The correct contacts when noise is only included on the Notch Intra Cellular Domain. **(a)** The percent of correct contacts when noise is on Notch and Delta (black) compared to when noise is only included on NICD (blue). The x-axis is normalized such that *σ*_*white*,*normalized*_ = *σ*_*white*_/200 (for noise on Notch and Delta) and *σ*_*white*,*normalized*_ = *σ*_*NICD*,*white*_/40 (for noise on NICD). **(b)** Same as (a) but the x-axis is normalized such that *σ*_*shot*,*normalized*_ = *σ*_*shot*_/20 (for noise on Notch and Delta) and *σ*_*shot*,*normalized*_ = *σ*_*NICD*,*shot*_/13 (for noise on NICD). **(c)** The percent of correct contacts when noise is only included on NICD as a function of the white noise amplitudes *σ*_*NICD*.*white*_. **(d)** Same as (c) except for shot noise. The results for both models (noise on N and D or noise on NICD) are averaged over 20 simulations after 1000hr of relaxation.
(TIF)

S23 Fig Transition waiting times computed based on the time it takes for a cell to travel from the Sender basin and cross the threshold into the Receiver basin as a function of Notch and Delta in the neighboring cells. (a) The average transition time for a Sender cell as a function of the Notch and Delta of its neighboring cells for σ_white_ = 1300. (b) Same as (a) but for σ_shot_ = 13. (c) Same as (a) but for σ_white_ = 2000. (d) Same as (a) but for σ_shot_ = 20. The simulations start with randomized initial conditions and the simulation is allowed to relax for 1000 hr. The times are averaged over 9000 hr of a single simulation and all cells in the lattice as a function of the Notch and Delta of the neighboring cells (N_EXT_ and D_EXT_).
(TIF)

S24 Fig Transition waiting times for the cell to transition from the Sender basin and into the Receiver basin as a function of the number of neighbors that are Receiver or Sender. (a) The average transition time as a function of the nearest neighbors that are Senders (<S_nn_>) and Receivers (<R_nn_>) for σ_white_ = 1300. (b) Same as (a) for σ_shot_ = 13. (c) same as (a) for σ_white_ = 2000. (d) Same as (a) for σ_shot_ = 20. The simulation is started with randomized initial lattices and relax for 1000 hr. The results are the average of all cells over 9000 hr of a single simulation.
(TIF)

S25 Fig Transition waiting times for the cell to transition from the Receiver basin and cross the threshold into the Sender basin as a function of the number of neighbors that are Receiver or Sender. (a) The average transition time as a function of the nearest neighbors that are Senders (<S_nn_>) and Receivers (<R_nn_>) for σ_white_ = 1300. (b) Same as (a) for σ_shot_ = 13. (c) same as (a) for σ_white_ = 2000. (d) Same as (a) for σ_shot_ = 20. The simulation is started with randomized initial lattices and relax for 1000 hr. The results are the average of all cells over 9000 hr of a single simulation.
(TIF)

S26 Fig The percent of correct contacts for different time steps compared to the chosen time step (dt = 0.1 hr). **(a)** The percent of correct contacts when white noise is present in the system with different time steps of 0.05 hr (green), 0.02 hr (orange), 0.2 hr (blue), and 0.1 hr (black, value used in main results). **(b)** Same as (a) but for shot noise. These results, especially for the zero noise case, show that the Euler method can be used for our results and a time step of dt = 0.1 hr is sufficient. The results are averaged over the last half of the simulation for 20 independent simulations of a 16x16 square multicell layer.
(TIF)

S27 Fig Confirmation randomness is implemented in the multicell model. **(a)** The randomness in the cellular level of notch when white noise is present. The standard deviation of the cellular Notch level was computed for every cell once the system relaxed for 1000 hr. The average value of this standard deviation is plotted showing an increase of Notch fluctuations as white noise levels increased which confirms randomness has been implemented in the system. **(b)** The same as (a) but for shot noise. **(c)** The same as (a) but for cellular Delta concentrations. **(d)** The same as (c) but for shot noise.
(TIF)
