# Supplementary material for: Stochastic fluctuations promote ordered pattern formation of cells in the Notch-Delta signaling pathway
Source: PLoS Comput Biol. 2022 Jul 21;18(7):e1010306. doi: 10.1371/journal.pcbi.1010306 (PMC9345490; doi:10.1371/journal.pcbi.1010306)
Supplement: S1 Text — A. Details of model and simulation for Notch-Delta switch. B. Details of the one cell Gillespie model. C. Simulation details of multicell model. (PDF) [file pcbi.1010306.s001.pdf]

S1 SIMULATION DETAILS

A. Details of model and simulation for Notch-Delta switch

In this work we begin with the one cell model exposed to a range of external Notch and Delta in the environment (N_{EXT} and D_{EXT}, respectively). For each (N_{EXT}, D_{EXT}) combination the cell relaxes until full equilibration.

To understand the Notch-Delta switch modeled by Eqs. (1)-(3), the initial value of N, D, and NICD in the cells was selected and the rate equations were numerically solved using the Euler-Maruyama method with a time step of dt=0.1 hr. The stochasticity of the system is computed by randomly sampling a value from a Gaussian Normal distribution and multiplying it by the corresponding amplitude (σ_{white} for white noise or $\sigma_{shot}\sqrt{X}$ for shot where X is either Notch or Delta in the cell). The random variable to be added to Notch, Eq (1), and Delta, Eq (2), are independent samples. Additionally, the simulation was constrained to ensure all molecular concentrations were strictly non-negative. Given that Notch and Delta vary on a range of 10^2-10^3 molecules, the amplitude of shot noise is approximately 10-100 times larger than the amplitude of white noise when $\sigma_{white}=\sigma_{shot}$.

Table A: Parameters of the stochastic rate equations for the Notch-Delta signaling pathway

Parameter	Value	Units
N_0	500	molecule/hr
D_0	1000	molecule/hr
k_c	5.0e-4	1/hr/molecule
k_t	5.0e-5	1/hr/molecule
γ	0.1	1/hr
γ_I	0.5	1/hr
I_0	200	Molecule

n_I	2
λ_N	2
λ_D	0

B. Details of the one cell Gillespie model

The Gillespie simulations were completed using the reactions and rates modeled with the zero noise equations of the one-cell system [Eq. (1)-(3), Table B in S1 Text]. The results are the average over the last 100,000 iterations of 10 independent Gillespie simulations starting from random initial conditions [N=U(0,6000), D =U(0,2000), and I = U(0,1000)] and completing 200,000 iterations. The values of N_{EXT} and D_{EXT} represent the exogenous ligands of the one cell system and were set to N_{EXT}=5000 and D_{EXT}=0 for Sender and N_{EXT}=0 and D_{EXT}=1500 for Receiver.

Table B: Reaction and propensity for the Gillespie model

Reaction	Propensity	Effect on copy number at time τ
$N + D_{ext} \overset{k_t}{\rightarrow} I$	$k_t N D_{ext}$	N-1, I+1
$D + N_{ext} \overset{k_t}{\rightarrow} I$	$k_t D N_{ext}$	D-1, I+1
$N + D \overset{k_c}{\rightarrow}$	$k_c N D$	N-1, D-1
$N \overset{\gamma}{\rightarrow}$	γN	N-1
$D \overset{\gamma}{\rightarrow}$	γD	D-1
$I \overset{\gamma_I}{\rightarrow}$	$\gamma_I I$	I-1
$N + nI \leftrightarrow NI_n$	$N_0 H(I, I_0, n, \lambda_N)$	N+1
$D + nI \leftrightarrow DI_n$	$D_0 H(I, I_0, n, \lambda_D)$	D+1

C. Simulation details of multicell model

The multicell system is a square lattice with periodic boundary conditions to control for cell-cell contact area and cell geometry. Thus, we can assume each cell contributes one quarter of its' Notch and Delta molecules to each of its four neighbors, implemented in our model as

$$N_{EXT} = \frac{1}{4} \sum_{i=1}^{4} N_i(t) , \qquad \text{(S1)}$$

for N_{EXT} in Eq. (2) and

$$D_{EXT} = \frac{1}{4} \sum_{i=1}^{4} D_i(t) , \qquad \text{(S2)}$$

for D_{EXT} in Eqs. (1) and (3).

To simulate the multicell model, the system was set to have a timestep of dt=0.1 hr and solved via the Euler-Maruyama method, as with the one cell model, and all molecular concentrations were constrained to be strictly non-negative. Additionally, we confirmed the results converged for all values of dt (see Fig. S26). In the stochastic models, the noise terms were calculated similar to the single cell system by randomly sampling from a Gaussian distribution using the random.normal function in NumPy. Additionally, a different value was sampled for every cell. The deterministic simulations were allowed to equilibrate for 5000 hr to reach a final pattern. The stochastic simulations had a relaxation period of 1000 hr. For each level of noise, a simulation length of 10000 hr, including the 1000 hr of relaxation, was completed for lattices starting from random initial conditions and averaged over 20 independent simulations unless noted. The non-random initial condition lattices were averaged over 20 different simulations with a length of 4000 hr after relaxation. We confirm the randomness in the systems increases as the amplitude of noise increases (Fig. S27).

Table C: The value of Notch, Delta, and Notch Intracellular Domain (NICD) in the Sender and Receiver cells in the deterministic multicell and two-cell models.

	Ligand/Receptor molecules		
State	Notch	Delta	NICD
Sender	567	1561	1
Receiver	5139	22	802

For a lattice with randomized initial conditions, the value of Notch, Delta and NICD is sampled from a random uniform distribution; the Notch level of the cell in the ith row and jth column is

$$N_{i,j}(t=0) = U(0, 10000) , \quad \text{(S3)}$$

the Delta level of the cell in the ith row and jth column is

$$D_{i,j}(t=0) = U(0, 10000) , \quad \text{(S4)}$$

and the NICD level of the cell in the ith row and jth column is

$$I_{i,j}(t=0) = U(0, 2000) . \quad \text{(S5)}$$

For systems with a specific initial configuration (e.g., checkerboard, nucleating, one quadrant of Receivers, etc.), the lattice is generated using only the values of Notch, Delta, and NICD of the deterministic two-cell model (Table C in S1 Text). If an initial lattice is said to have a specific percentage of mistakes, then a specific percentage of cells in a checkerboard lattice were randomly selected and perturbed away from a perfect checkerboard. These cells have their values of Notch, Delta, and NICD replaced by randomly sampling a new value from a uniform distribution; the updated value of Notch in the cell is

$$N_{new}(t=0) = U(0, 10000) , \quad \text{(S6)}$$

the updated value of Delta is

$$D_{new}(t=0) = U(0, 10000) , \quad \text{(S7)}$$

and the updated value of NICD is

$$I_{new}(t=0) = U(0, 10000) . \tag{S8}$$

Lastly, if a checkerboard has been perturbed by a standard deviation, then this lattice was modified from checkerboard. For each cell in the checkerboard lattice, a value sampled from a Gaussian with mean $\mu = 0$, and standard deviation B, was added to the value of Notch, Delta, and NICD (sampled from independent distributions); for Sender cells

$$N_{new}(t=0) = N_{Sender} + BNormal(0, 1) , \tag{S9}$$

$$D_{new}(t=0) = D_{Sender} + BNormal(0, 1) , \tag{S10}$$

and

$$I_{new}(t=0) = I_{Sender} + BNormal(0, 1) . \tag{S11}$$
