# Supplementary figures and images for: Stochastic fluctuations promote ordered pattern formation of cells in the Notch-Delta signaling pathway
Source: PLoS Comput Biol. 2022 Jul 21;18(7):e1010306. doi: 10.1371/journal.pcbi.1010306 (PMC9345490; doi:10.1371/journal.pcbi.1010306)

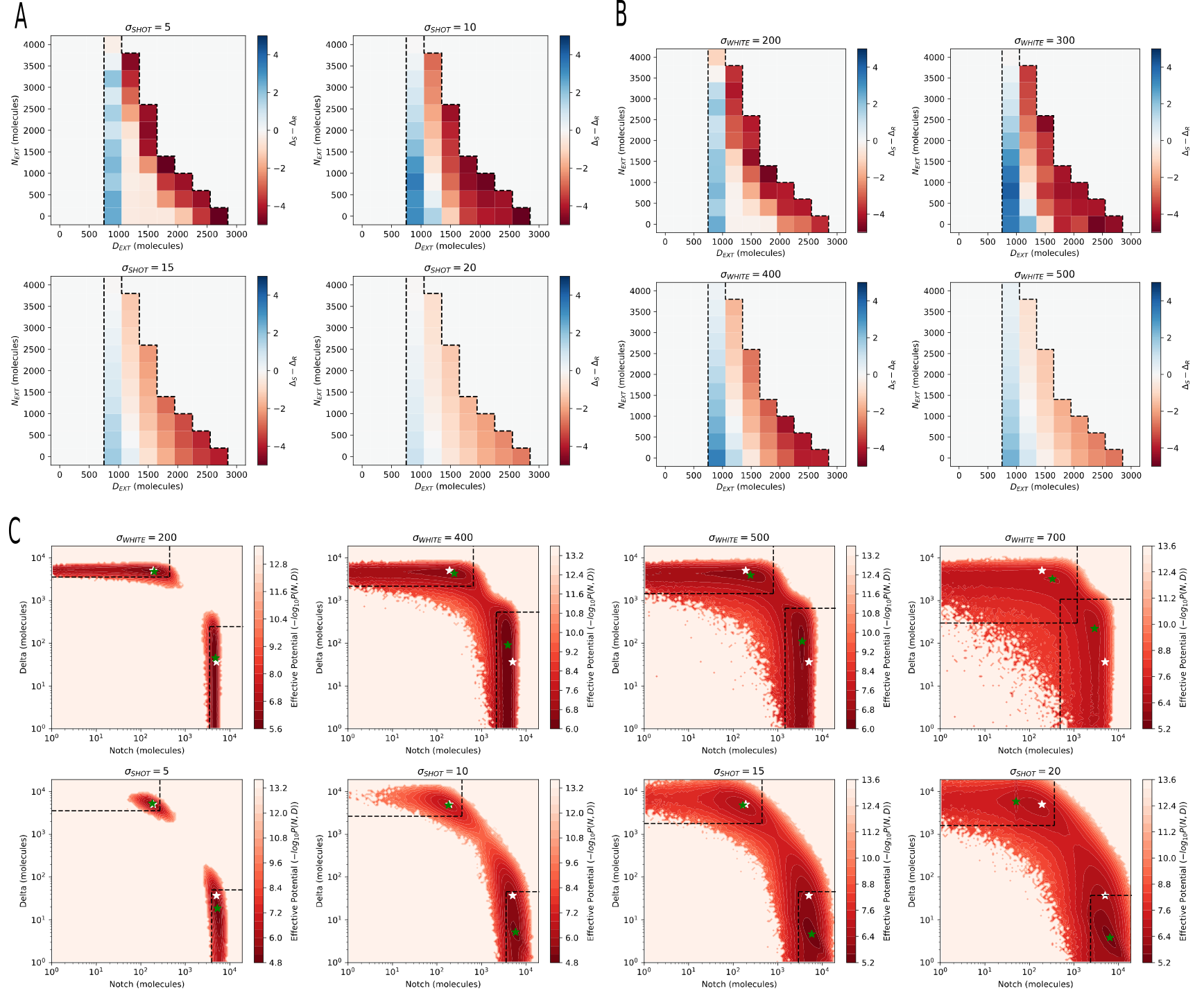

Supplement: S1 Fig — (a) Difference between barrier height from Sender to Receiver state (ΔS) and barrier height from Receiver to Sender state (ΔR) as a function of external Delta ligands (DEXT, x-axis) and Notch receptors (NEXT, y-axis). Four panels show increasing levels of shot noise. (b) Same as (a) for varying levels of white noise. (c) Pseudopotential landscape for increasing levels of white noise (top) and shot noise (bottom). White starred dots highlight the location of the stable fixed points of the corresponding deterministic model, while green starred points show the location of the landscape minima. For panel (c), DEXT and NEXT have the same values as in Fig 1(b). (TIF) [file pcbi.1010306.s004.tif]

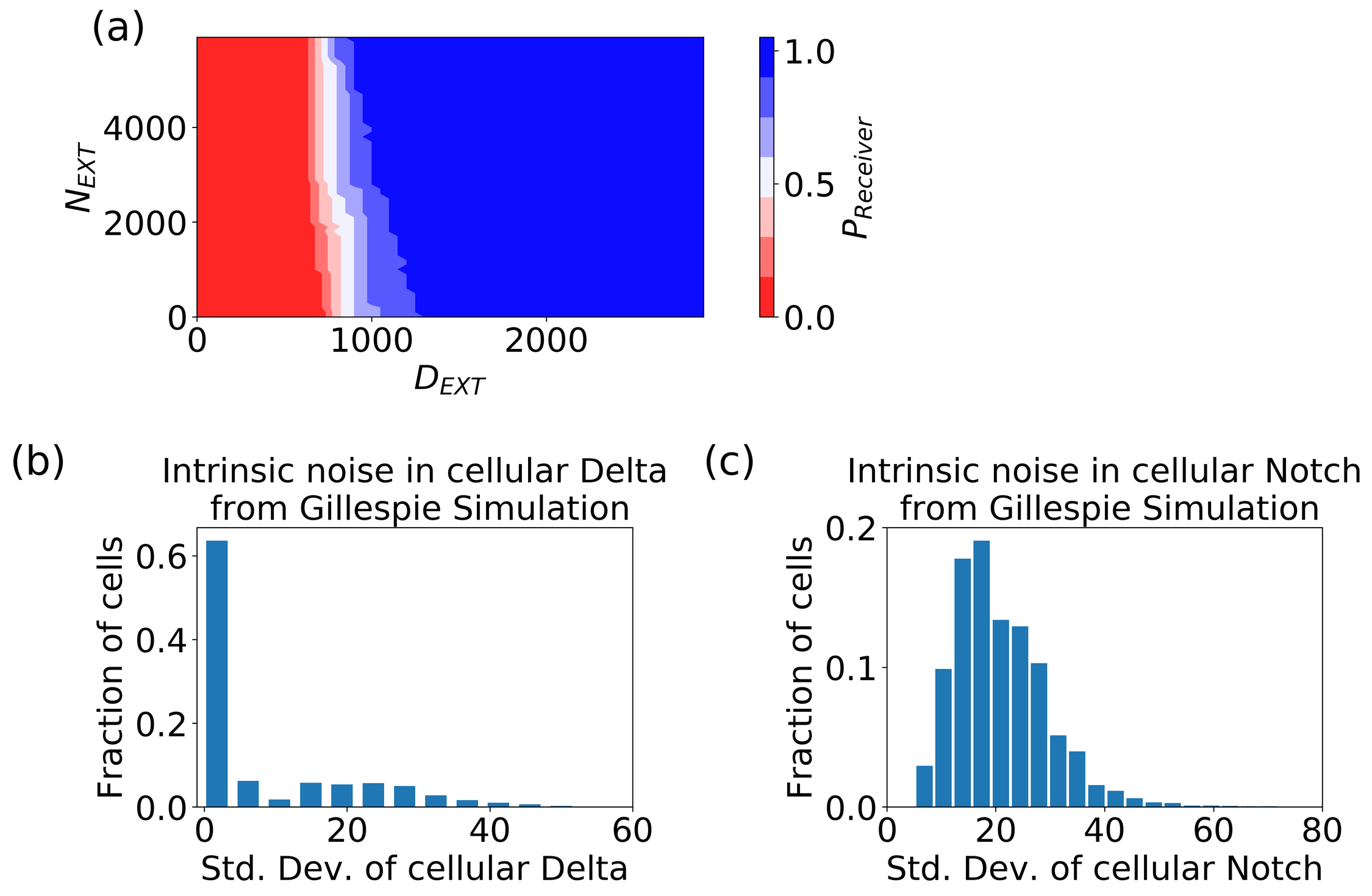

Supplement: S2 Fig — (a) The probability of a cell being a Receiver for a particular level of exogenous Delta and Notch. The results of ten simulations were averaged to develop this phase plane. (b) c standard deviation of Delta in a cell for all NEXT and DEXT pairs of (a) corresponding to the intrinsic noise of cellular Delta. (c) The same as (b) for cellular Notch. (TIF) [file pcbi.1010306.s005.tif]

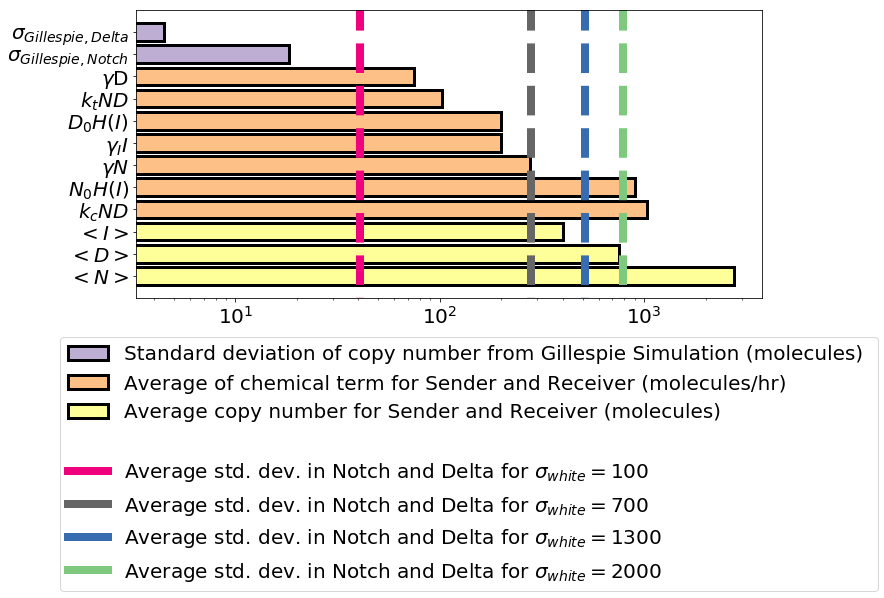

Supplement: S3 Fig — The purple bars are the intrinsic noise calculated from Gillespie simulations and have a magnitude of about 10. The orange bars are the chemical terms averaged over the Sender and Receiver values and have a magnitude of about 102. The yellow bars are the average N, D, and I copy numbers with magnitude averaging 103. The bars show approximately which levels of added noise in the system are comparable to various categories (intrinsic noise, chemical terms, or cellular concentrations). (TIF) [file pcbi.1010306.s006.tif]

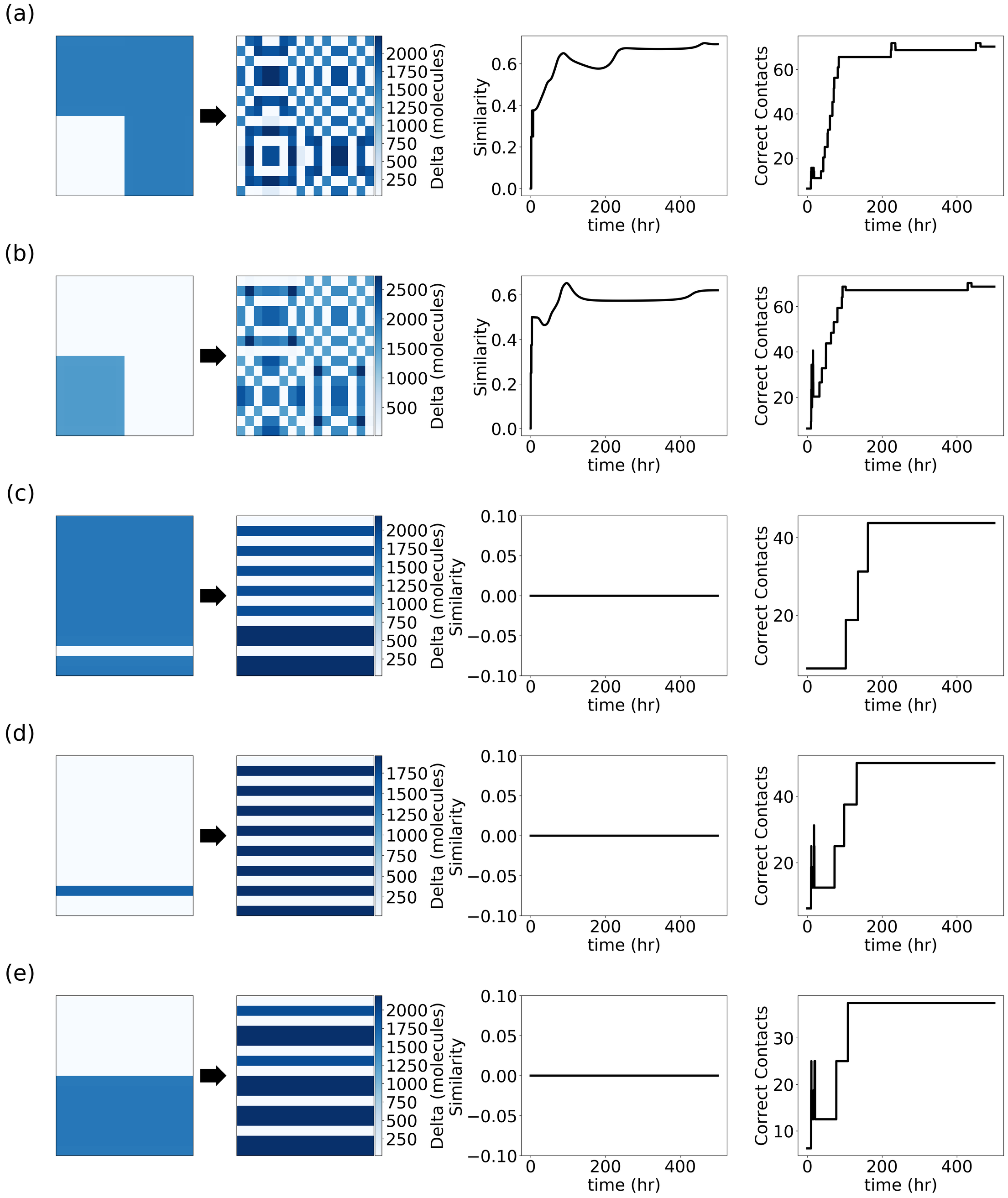

Supplement: S4 Fig — The initial pattern (left), final pattern (middle left), similarity (middle right), and percent of correct contacts (right) as a function of time for a quadrant of Senders, a quadrant of Receivers, a line of Senders, a line of Receivers, and half Senders and half Receivers, (a)-(f) respectively. (TIF) [file pcbi.1010306.s007.tif]

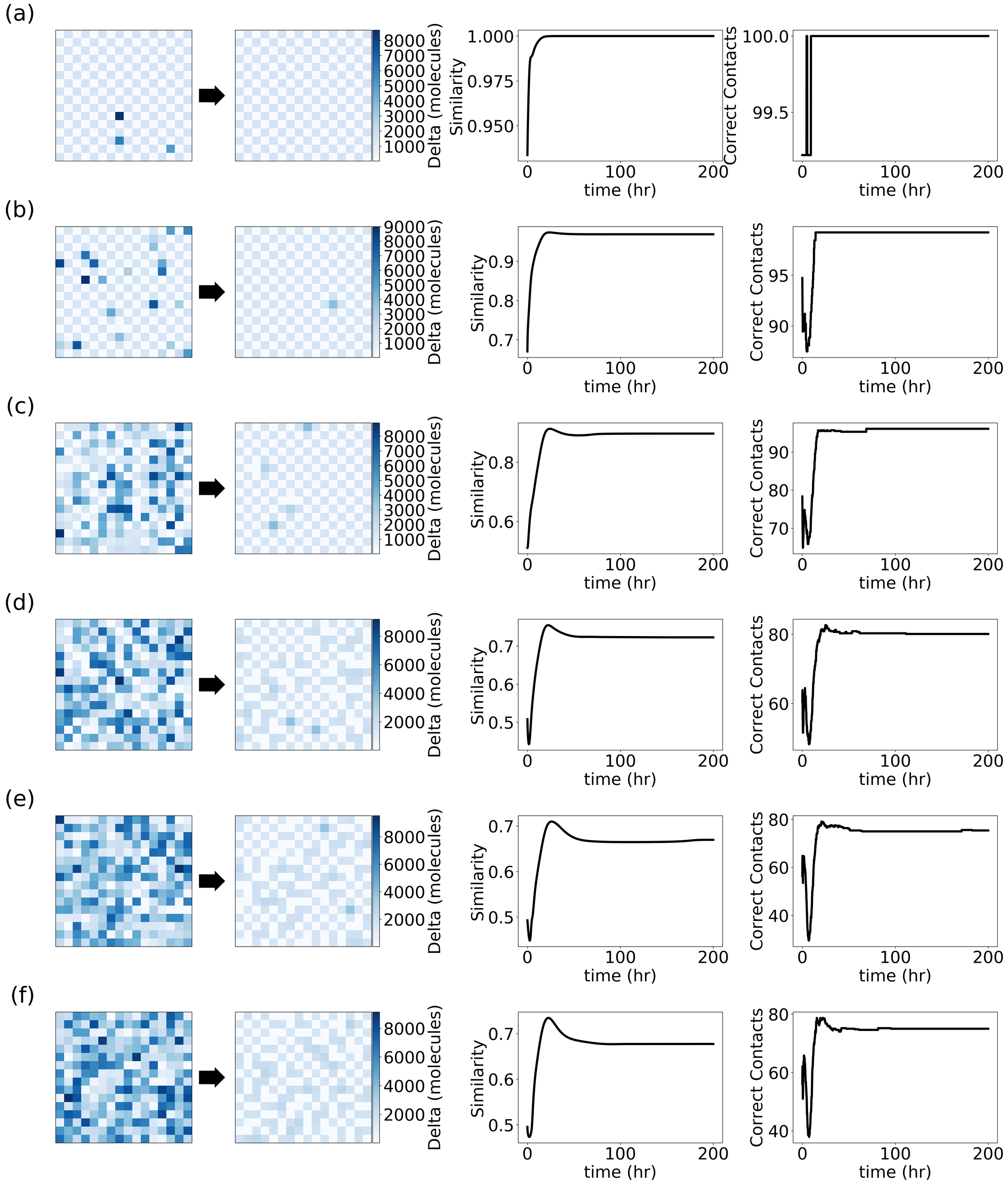

Supplement: S5 Fig — Example of initial pattern (left), final pattern (middle left), similarity metric (middle right), and percent correct contacts (right) as a function of time for deterministic simulations for initial checkerboard lattices with discrete perturbations. The number of initial mistakes in the 256-cell lattice are 3 (1.2%), 26 (10.2%), 128 (50%), 192 (75%), 243 (94.9%), and 253 (98.8%) mistakes for (a)-(f), respectively. (TIF) [file pcbi.1010306.s008.tif]

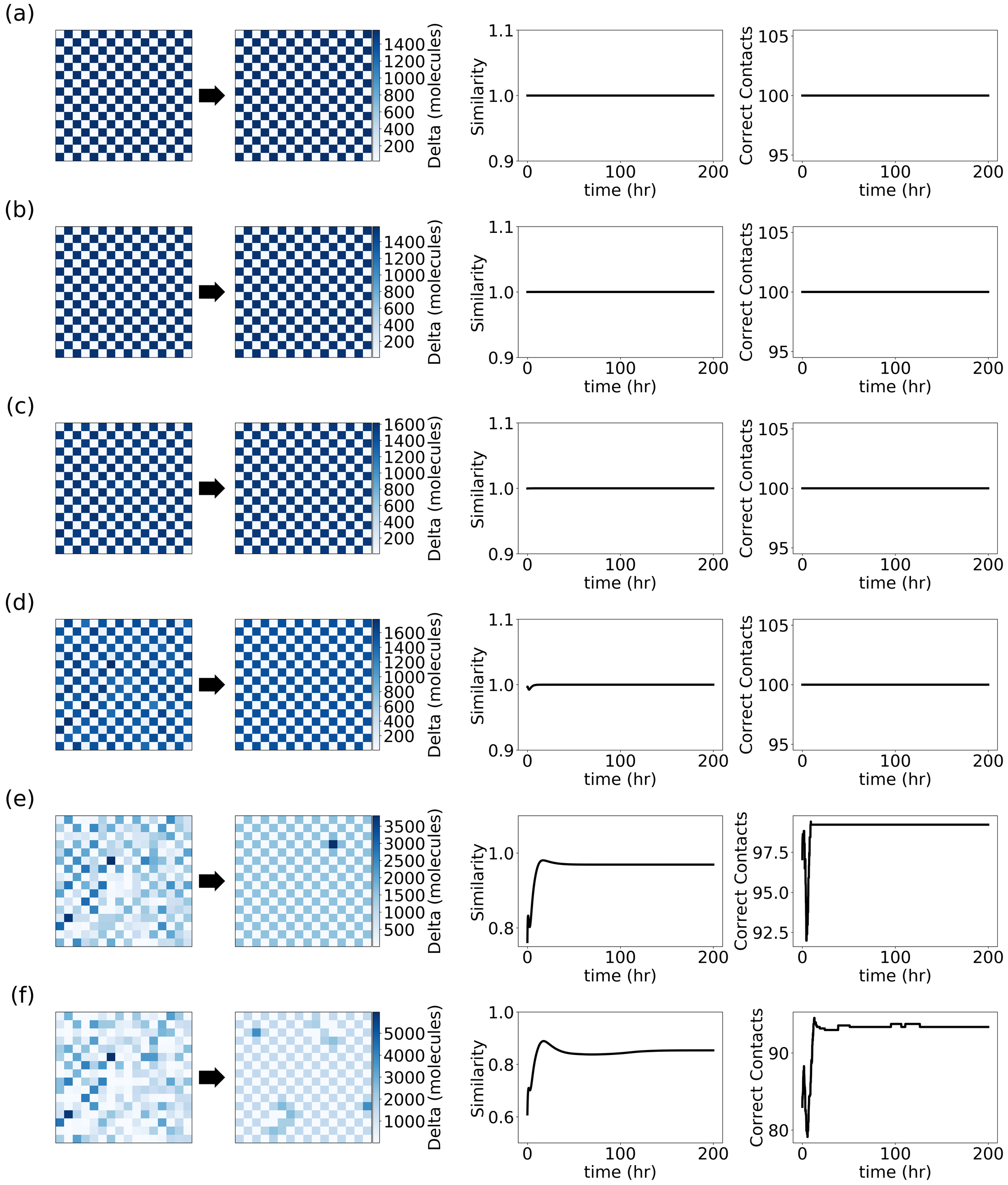

Supplement: S6 Fig — Example of deterministic results for initial checkerboard lattices with continuous perturbations showing the initial pattern (left), final pattern (middle left), similarity metric (middle right), and percent of correct contacts (right) as a function of time. The initial conditions for the simulations had an added Gaussian random variable of mean μ = 0 and standard deviation of B = 1, 10, 25, 100, 1000, and 2000 (a-f, respectively). (TIF) [file pcbi.1010306.s009.tif]

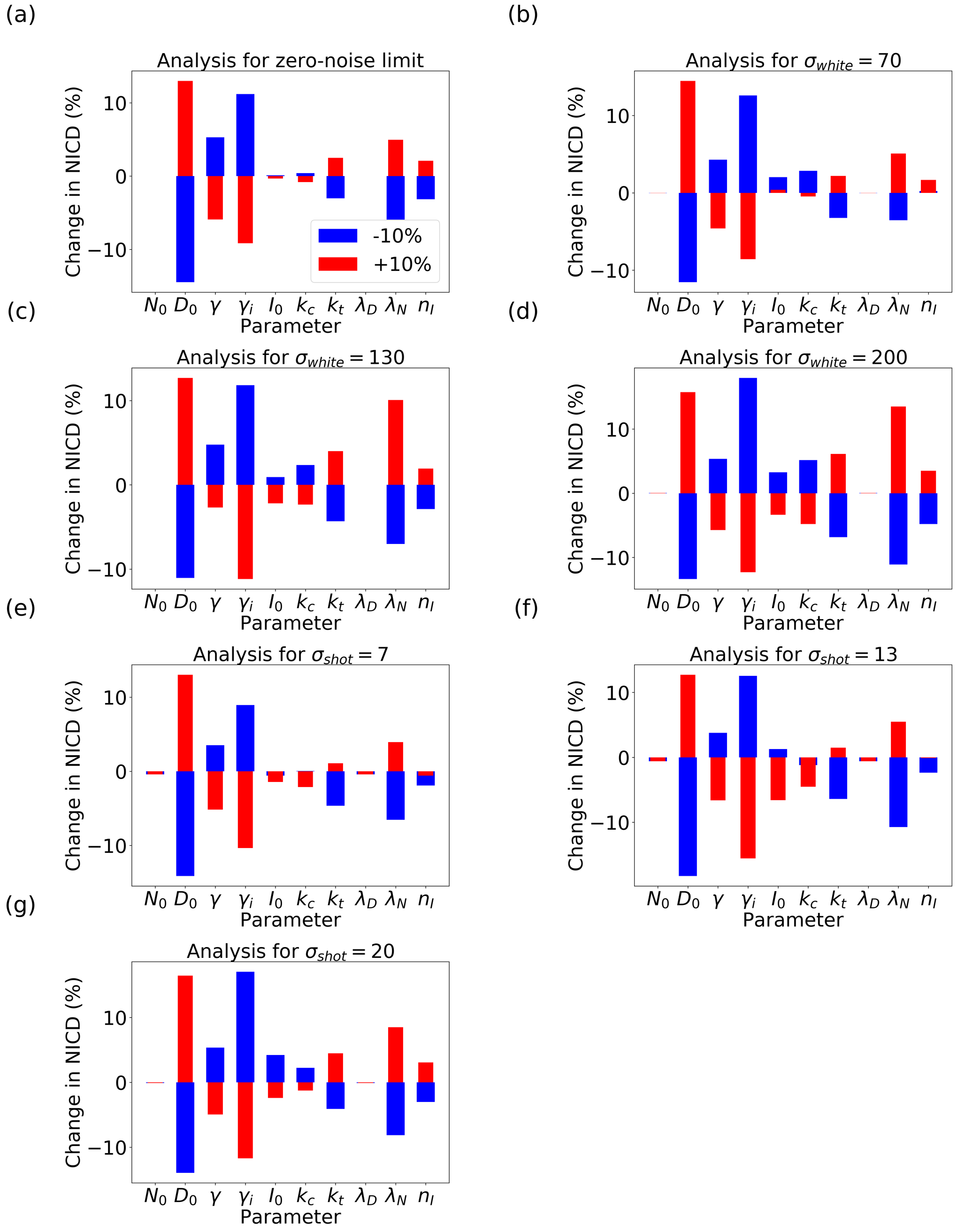

Supplement: S7 Fig — The difference in NICD when a single parameter is changed compared to model with no changed parameters. The results are the average for a multicell system once the system has equilibrated. (a) For deterministic model showing robustness. (b)-(d) Same as (a) but for the stochastic model with white noise at σwhite = 700, 1300, and 2000. (e-g) Same as (a) but for stochastic model where σshot = 7,13,20. (TIF) [file pcbi.1010306.s010.tif]

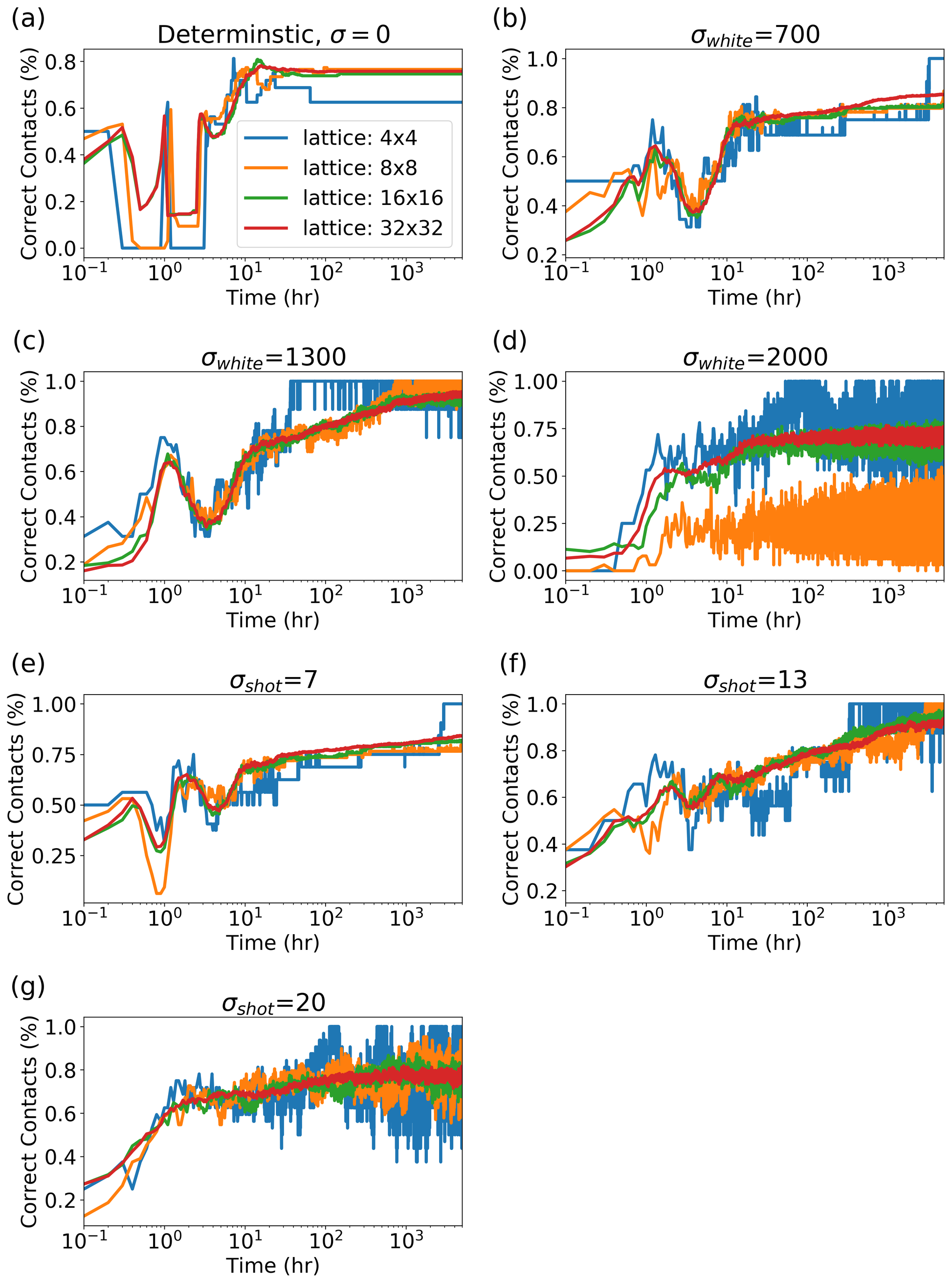

Supplement: S8 Fig — (a) The correct contacts for deterministic multicell simulations with a square cell length of 4 (blue), 8 (orange), 16 (green), and 32 (red). The systems with lattice length larger than 4 converge to the same solution. (b-d) Same as (a) but for stochastic model where σwhite = 700, 1300, and 2000. (e-g) Same as (a) but for stochastic model where σshot = 7,13,20. These results show that lattices of size 16 and 32 have converged to the same result, therefore we can simply our calculations and use the smaller lattice of length 16 cells. (TIF) [file pcbi.1010306.s011.tif]

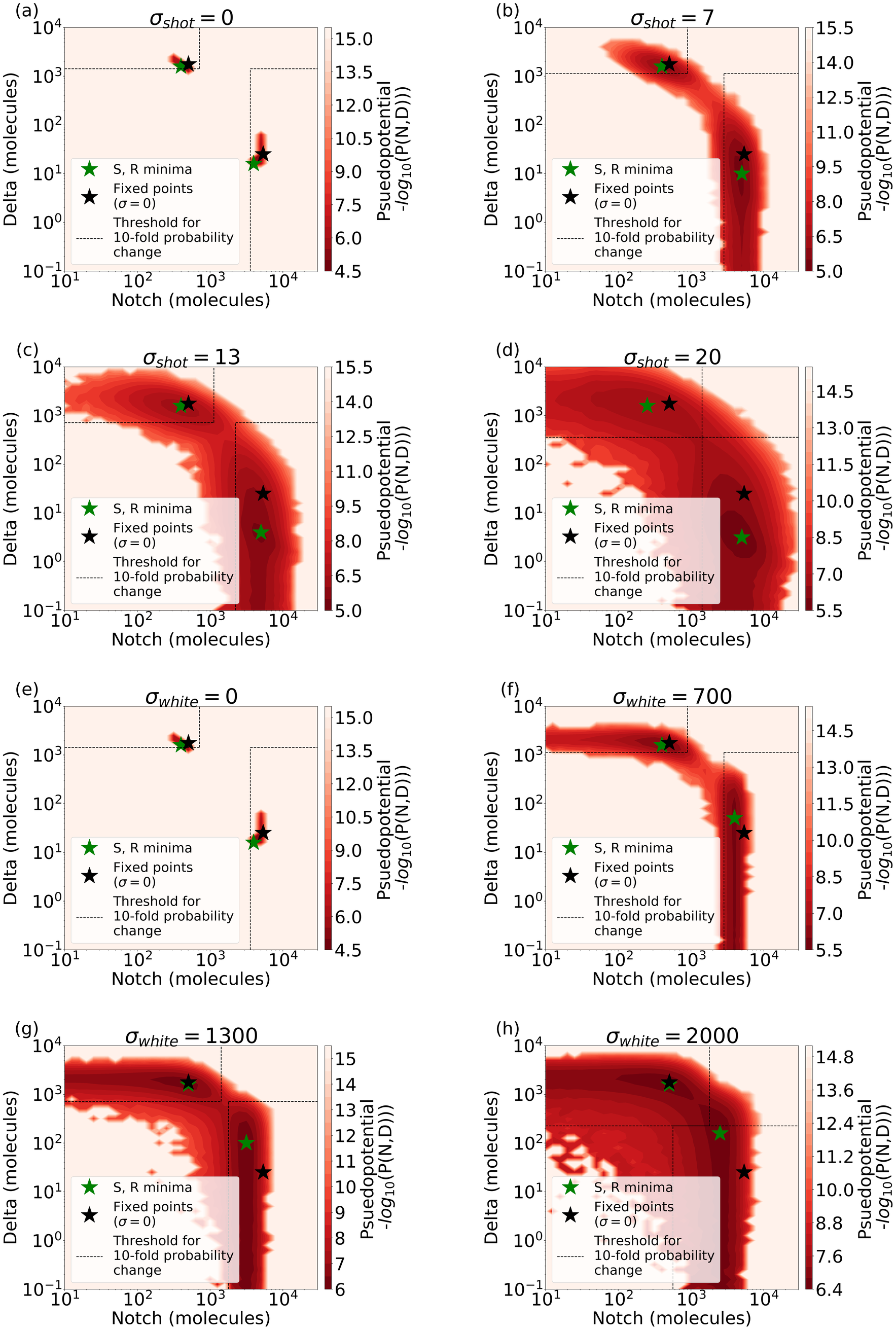

Supplement: S9 Fig — The pseudopotential landscapes U = -log10 P(N,D), where P(N,D) is the probability of a cell having a level of Notch and Delta equal to N and D, respectively. The green stars are the location of the pseudopotential minima, while the black stars represent the location of the Sender and Receiver states in a perfect checkerboard pattern without noise. The dotted lines depict the thresholds for the Sender and Receiver states. If the value of Notch and Delta of the cell are within a 10-fold difference from the Notch and Delta of the closest minima, then the cell is considered as in that basin of attraction. (a)-(d) Pseudopotential landscapes with stochastic fluctuation amplitudes of σshot = 0, 7, 13, and 20. (e)-(f) Pseudopotential landscapes with σwhite = 0, 700, 1300, and 2000. (TIF) [file pcbi.1010306.s012.tif]

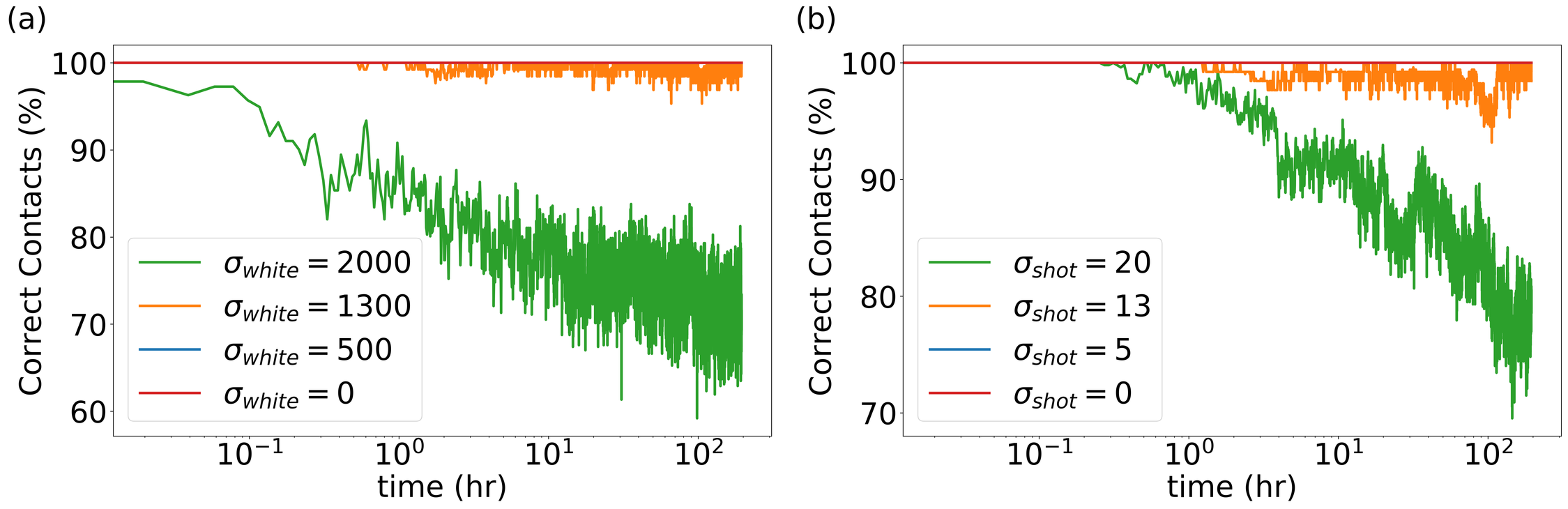

Supplement: S10 Fig — (a) The correct contacts or deterministic case (red), σwhite = 50 (green), σwhite = 130 (orange), and σwhite = 200(blue). (b) The correct contacts for the deterministic case (red), σshot = 5 (green), σshot = 13 (orange), and σshot = 20 (blue). (TIF) [file pcbi.1010306.s013.tif]

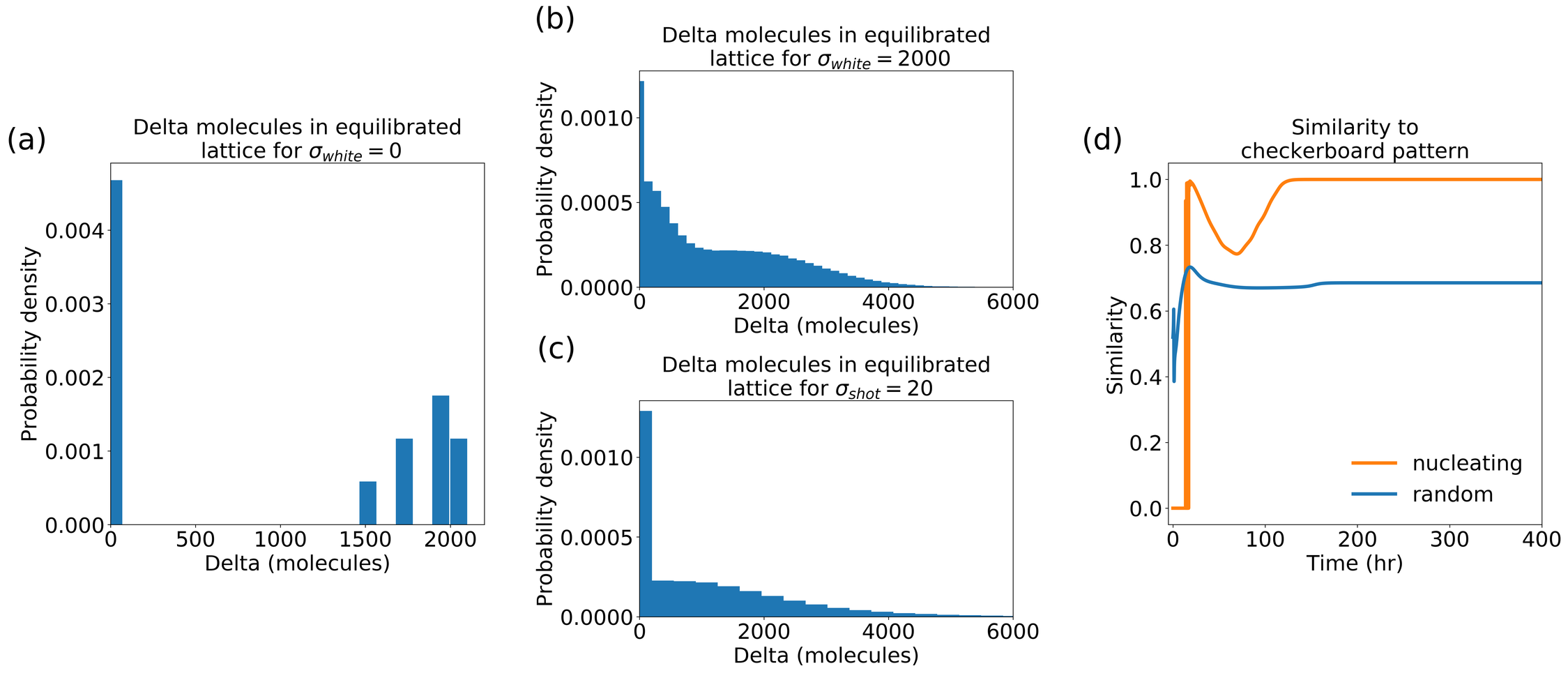

Supplement: S11 Fig — (a) The probability density of Delta molecules in all cells after the system has relaxed starting from randomized initial conditions with no applied noise for a single independent simulation. This density shows two distinct cell populations, Sender on the right and Receiver on the left. (b) Same as (a) but for σwhite = 2000 showing the populations are no longer distinct. (c) Same as (a) but for σwhite = 20. For (a), the distribution is computed for a representative sample of the cells in the lattice (6.25%) for a simulation starting from randomized initial conditions after equilibration to steady state (1000 hr). For (b) and (c) the distributions are computed for the sample of cells after the system has relaxed for 1000 hr. (d) The similarity to checkerboard starting from randomized initial conditions (blue) and the nucleating initial condition (lattice with one Sender and all other Receivers). Both are deterministic simulations showing the first 400 hr during which the simulation equilibrates to its final pattern (frustrated or checkerboard, respectively). (TIF) [file pcbi.1010306.s014.tif]

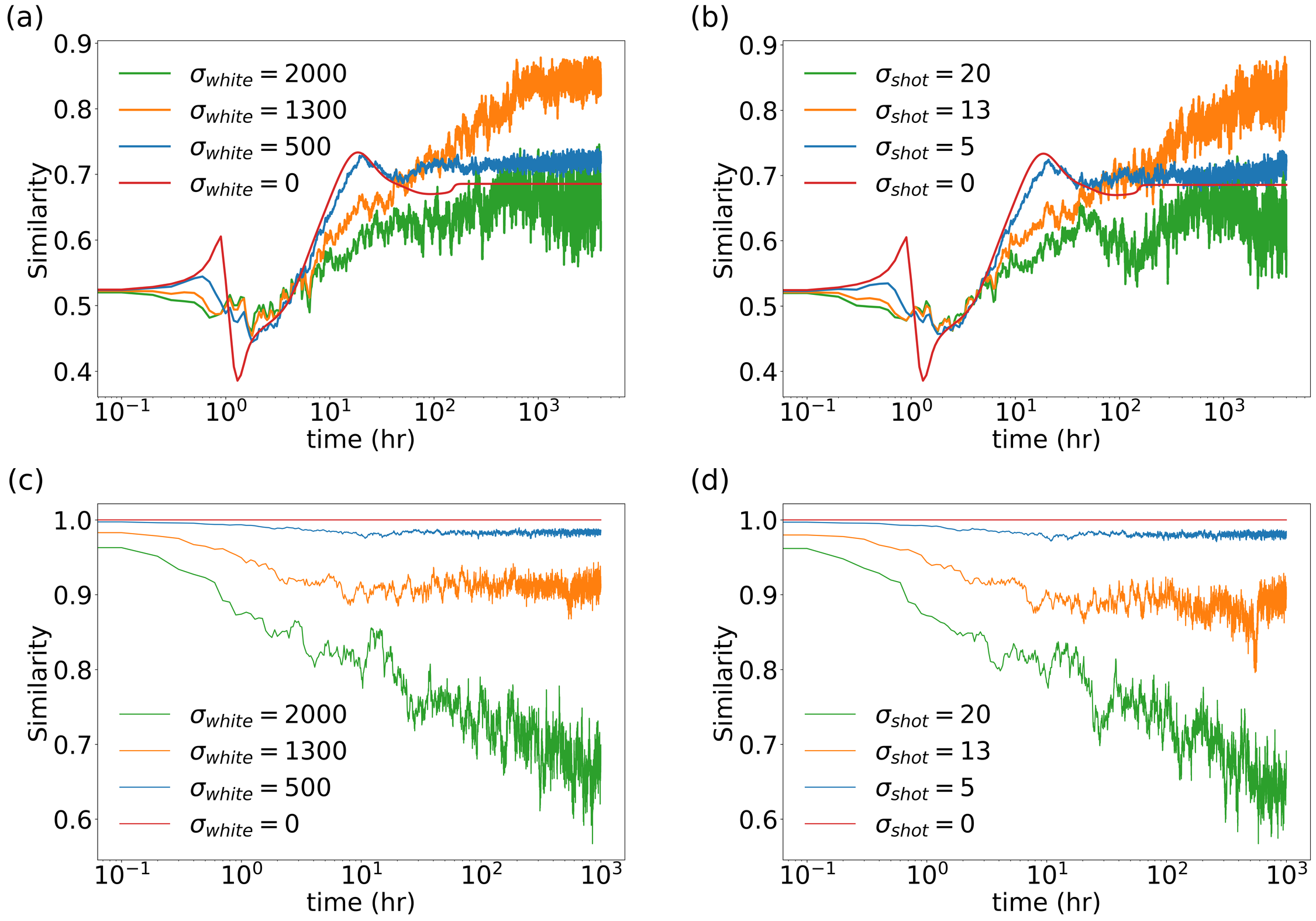

Supplement: S12 Fig — (a) The similarity as a function of time for randomized initial conditions showing the correlation of Delta in the cells throughout the lattice for the deterministic case (red, σwhite = 0), low noise case (green, σwhite = 500), medium noise case (orange σwhite = 1300), and high noise (blue, σwhite = 2000). (b) Same as (a) but with corresponding levels of shot noise (σshot = 0, 5, 13, 20). (c) Same as (a) but for checkerboard initial conditions. (d) Same as (b) but for checkerboard initial conditions. (TIF) [file pcbi.1010306.s015.tif]

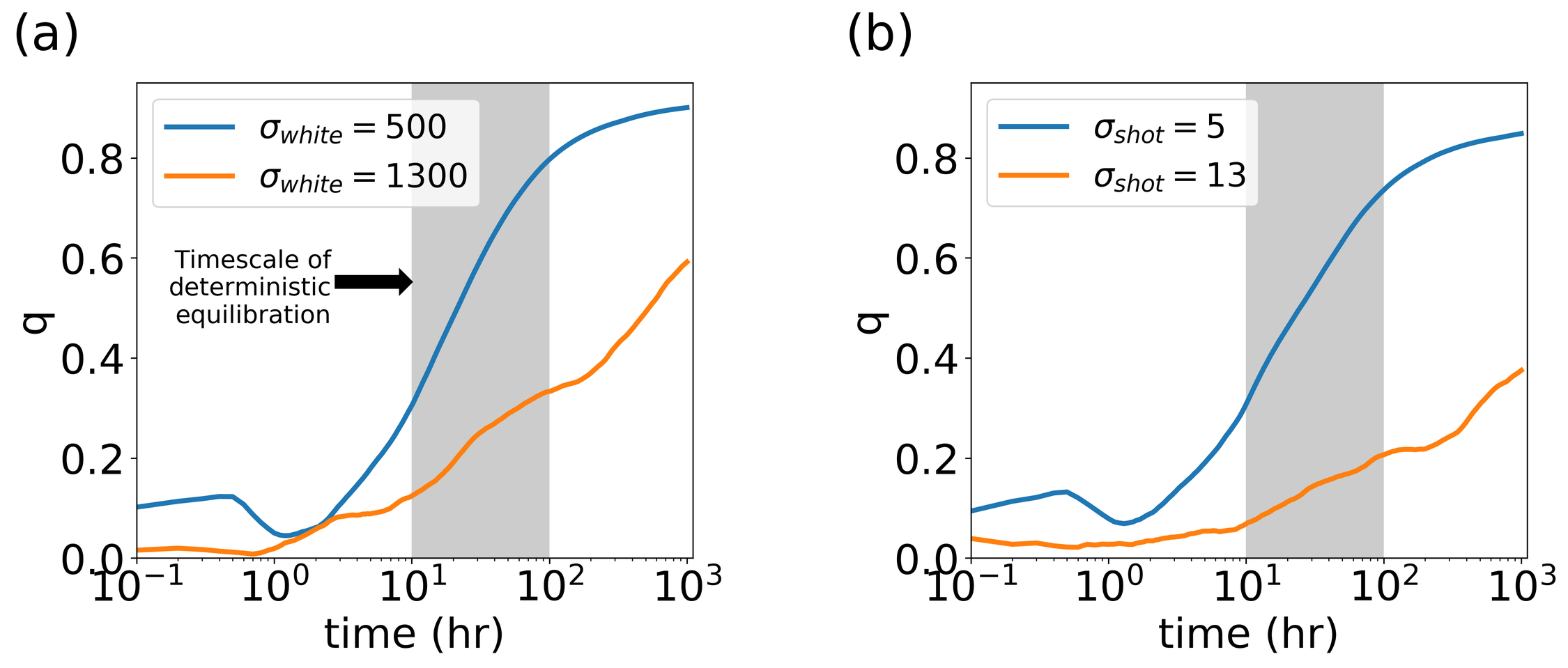

Supplement: S13 Fig — (a) The time averaged autocorrelation function (q) comparing lattices with the final lattice pattern of the system in a stochastic system with σwhite = 500 and σwhite = 1300. The pattern begins to converge toward the final lattice pattern at around 10 hr. (b) In a stochastic system with σshot = 5 and σshot = 13, the time averaged autocorrelation function comparing lattices to the final lattice. The lattices converge towards a more ordered system starting at about 10 hr. The gray area is the typical timescale for the Notch system to equilibrate. The blue curves (intermediate noise) show a large slope in the grey region corresponding to fast equilibration driven by chemical kinetics and a smaller slope on the rightmost region of the plots corresponding to the error-correction driven by stochastic fluctuations. (TIF) [file pcbi.1010306.s016.tif]

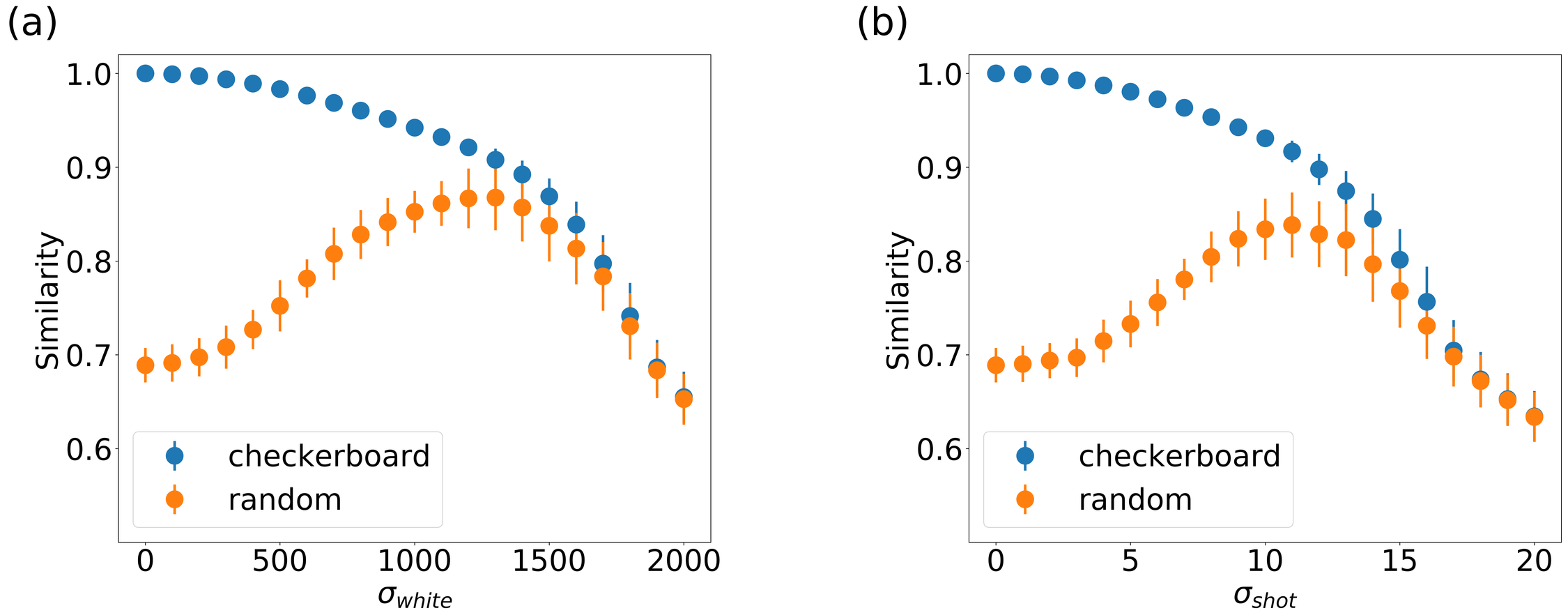

Supplement: S14 Fig — (a) The similarity metric for randomized initial conditions (orange) and checkerboard initial conditions (blue) as a function of increasing white noise amplitude. (b) The same as (a) for shot noise. For all simulations there were 1000 hr allowed for the system to relax. For the randomized initial conditions, the results were averaged over 9000 hr for 20 independent simulations. The initially checkerboard system was averaged over 400 hr for 20 distinct simulations. The trends seen in the similarity metric mimic those exhibited by the correct contacts as a function of white or shot noise. While the similarity metric drops below S = 1 for the checkerboard initial condition more quickly than the fraction of correct contacts changes (see Fig 4), the steady decrease in the similarity metric is representative of the modulation and a loss of separation in the Delta values of the Sender and Receiver cell (see again S7 Fig). (TIF) [file pcbi.1010306.s017.tif]

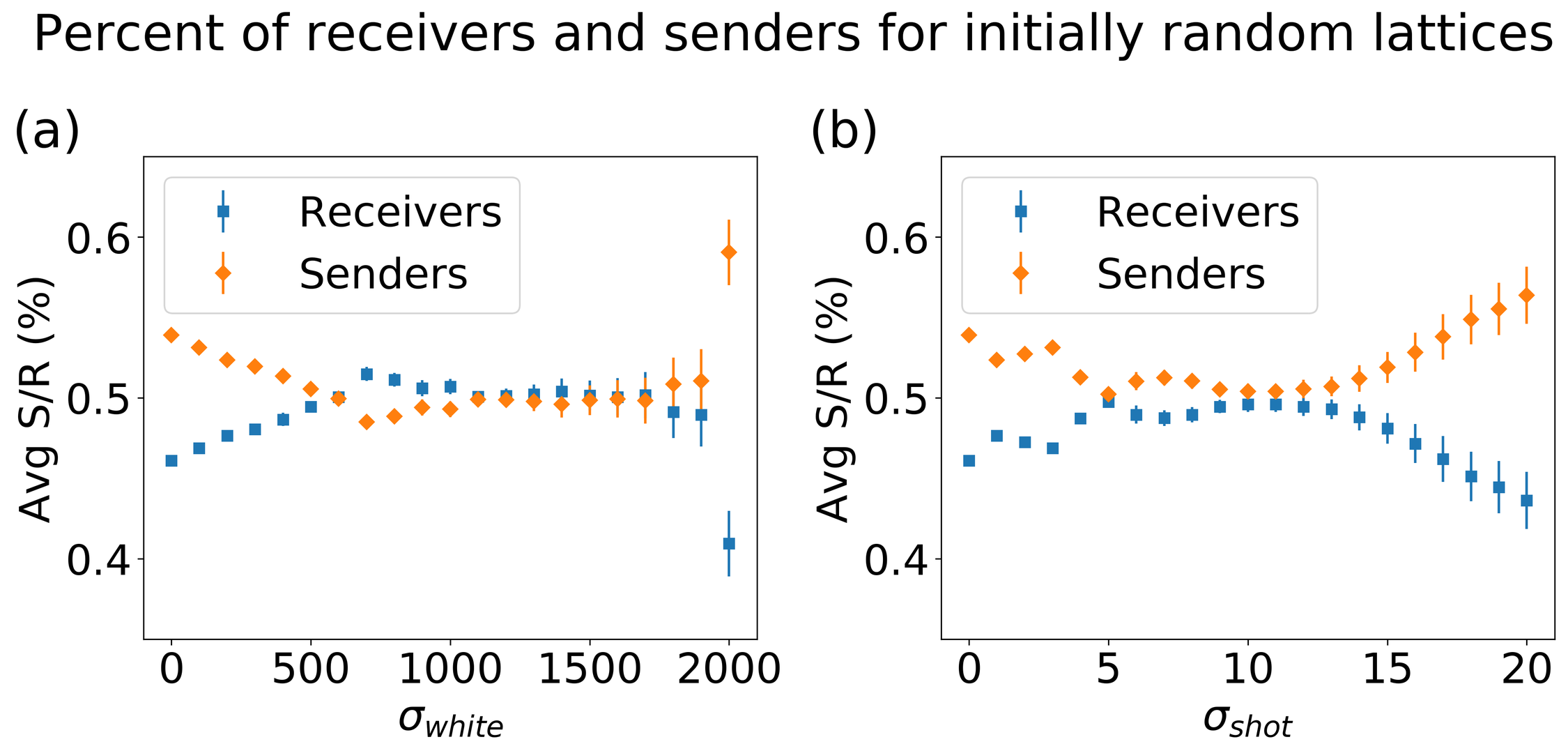

Supplement: S15 Fig — (a) The average percent of Receivers and Senders as a function of increasing white noise amplitude. (b) same as (a) but for shot noise. The simulations are for lattices with randomized initial conditions. The lattices were allowed to relax for 1000 hr and then the results were averaged over the last 9000 hr of the 20 independent simulations. Throughout the intermediate noise regime nearly half of the cells are in the Sender state and the other half are in the Receiver state, confirming systems in this regime has the correct ratio of Senders to Receivers to achieve a checkerboard patterning. (TIF) [file pcbi.1010306.s018.tif]

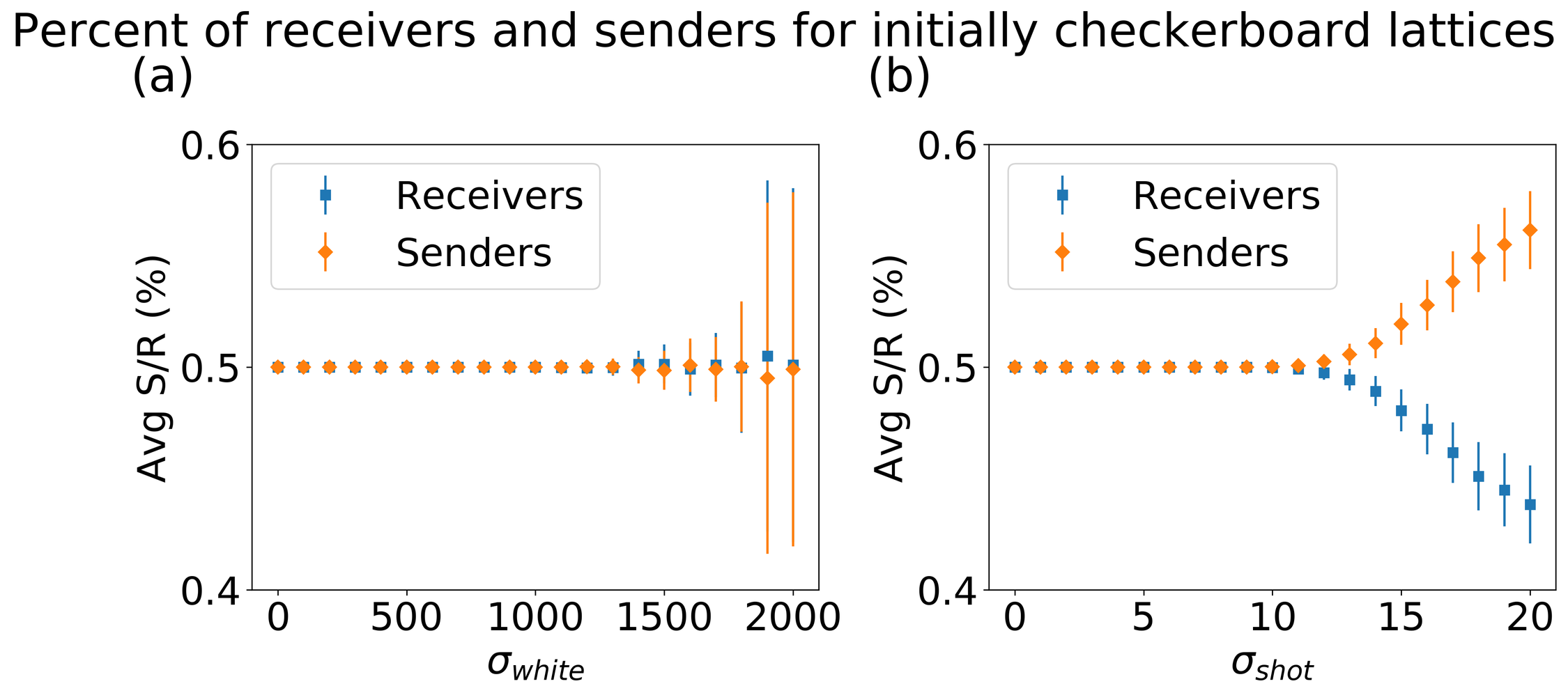

Supplement: S16 Fig — (a) The average percent of Receivers and Senders as a function of increasing white noise amplitude for initially checkerboard lattices. (b) same as (a) but for shot noise. The lattices were allowed to relax for 1000 hr and then the results were averaged over the last 4000 hr of 20 distinct simulations. Throughout the low and intermediate noise regime nearly half of the cells are in the Sender state and the other half are in the Receiver state, confirming the systems have the correct ratio of Senders to Receivers to achieve a checkerboard patterning. (TIF) [file pcbi.1010306.s019.tif]

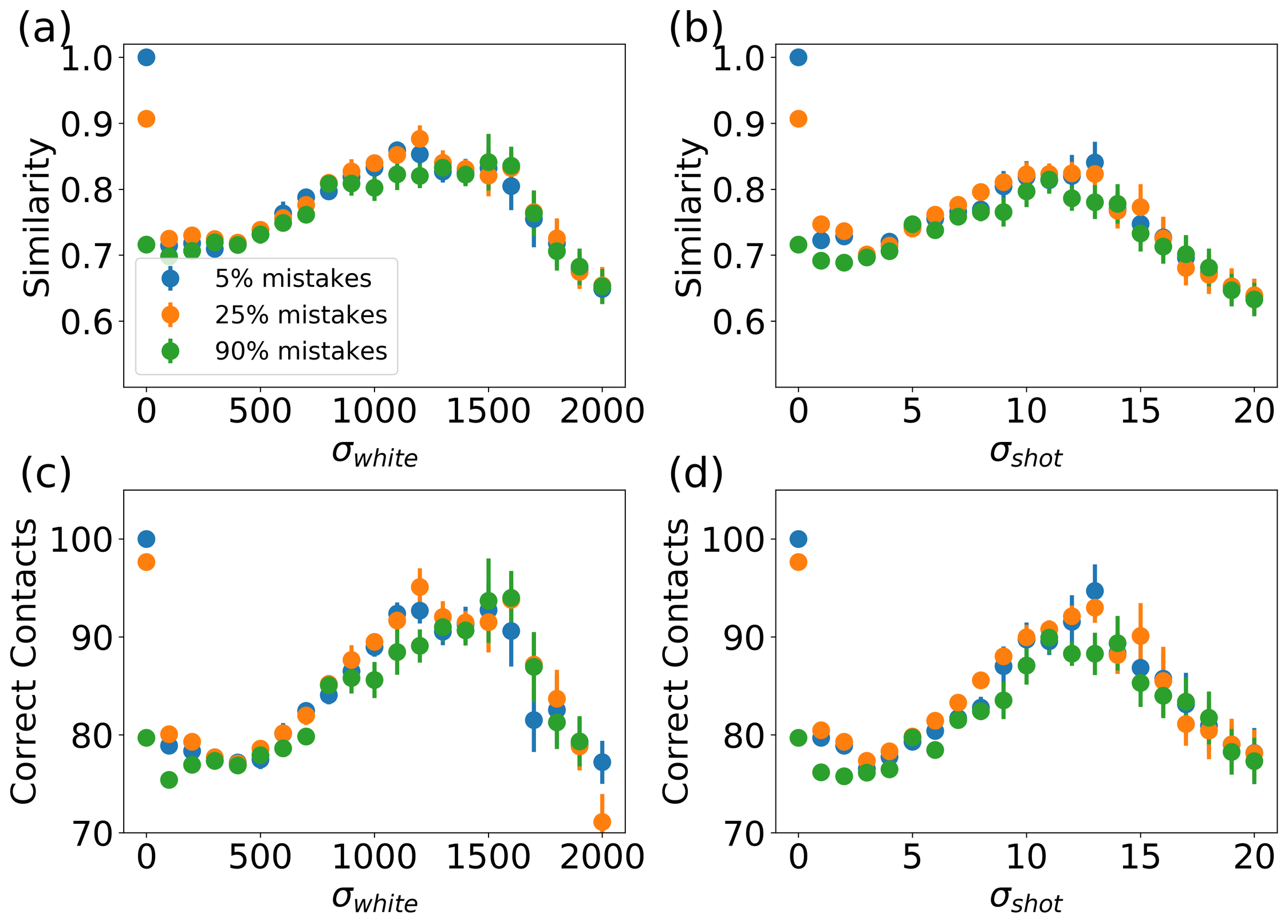

Supplement: S17 Fig — (a) The similarity as a function of increasing white noise amplitude for checkerboard lattices with discrete perturbations of 13, 64, and 230 mistakes (corresponding to 5%, 25%, and 90% of mistakes in the lattice). (b) same as a but for shot noise. (c) same as (a) but for correct contacts as white noise increases. (d) same as (c) but for shot noise. The simulations were allowed to relax for 1000 hr and then averaged over the last 4000 hr of 20 simulations with different initial conditions. The checkerboard lattices with discrete perturbations follow a trend analogous to the randomized initial lattice where the systems are most ordered in the intermediate regime. (TIF) [file pcbi.1010306.s020.tif]

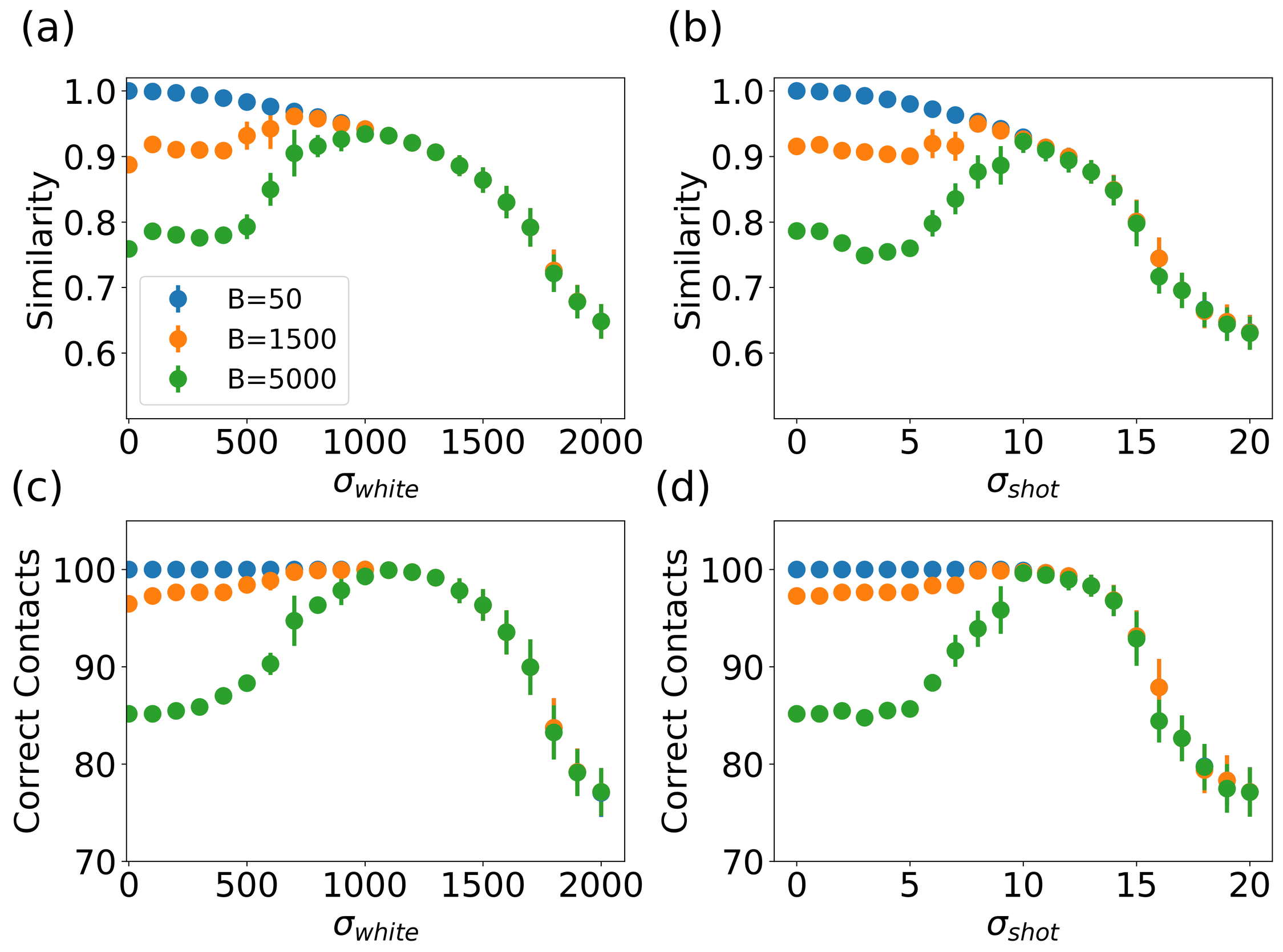

Supplement: S18 Fig — (a) The similarity metric with increasing stochastic fluctuations from white noise for a checkerboard initial condition with an added Gaussian random variable of mean μ = 0 and standard deviation of B = 50, 1500, and 5000. (b) same as (a) but for shot noise. (c) Same as (a) but for percent of correct contacts as a function of increasing white noise. (d) Same as (c) but for shot noise. The simulations were allowed to relax for 1000 hr and then averaged over the last 4000 hr of 20 simulations with different initial conditions. Interestingly, in the presence of stochastic fluctuations, checkerboard lattices with continuous perturbations to all cells behave comparably to the checkerboard initial condition when perturbations are small and correspond to the randomized initial conditions when perturbations are larger. (TIF) [file pcbi.1010306.s021.tif]

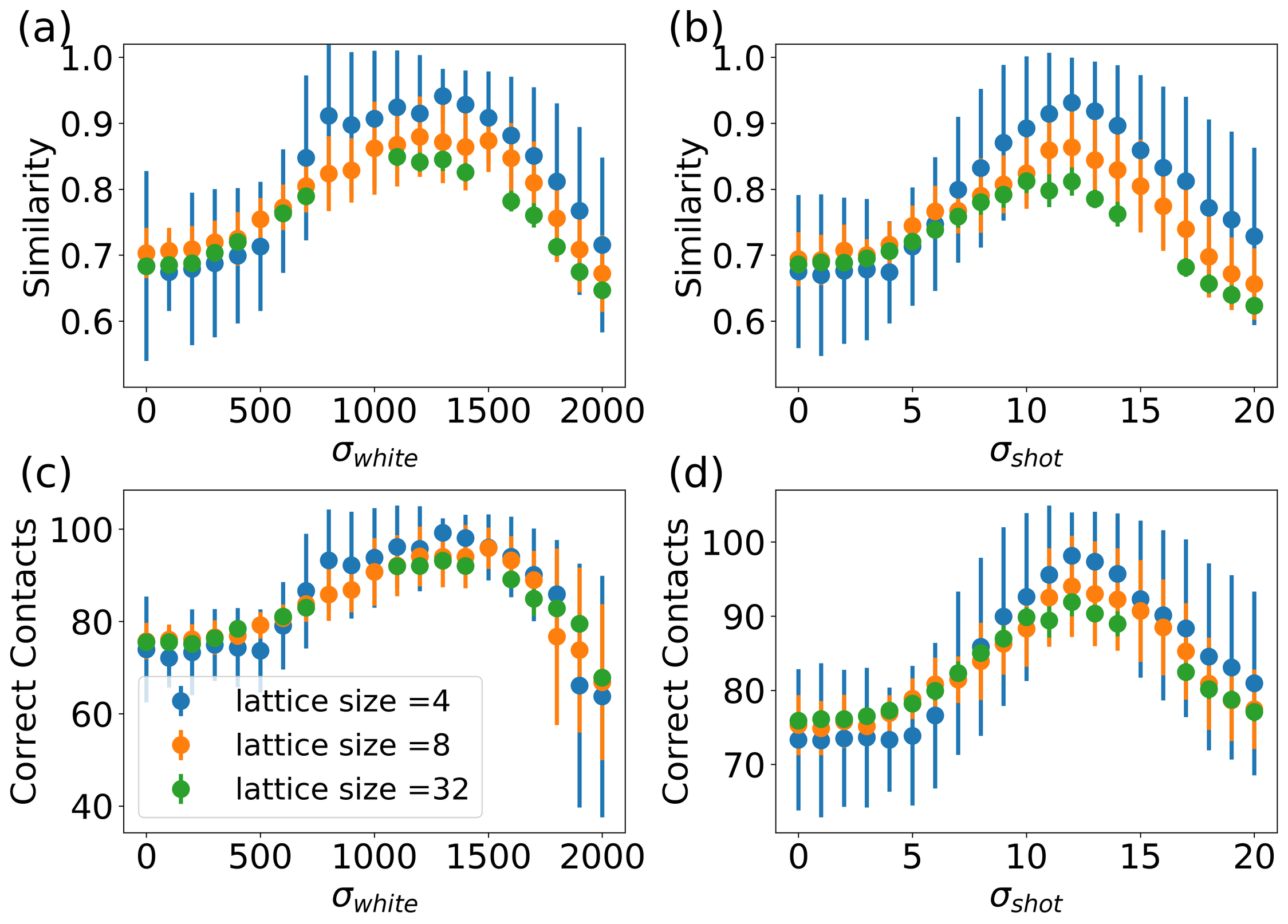

Supplement: S19 Fig — (a) The similarity metric as a function of white noise for square lattices of length 4, 8, and 32 (square lattice of length 16 is in the main text). (b) Same as (a) but for shot noise. (c) same as (a) but for correct contacts as a function of increasing white noise. (d) Same as (c) for shot noise. There were 20 simulations starting with different initial conditions and averaged over 4000 hr after they were allowed to relax for 1000 hr. The response to noise is consistently observed across lattice sizes, with the trend being more robust as the size of the lattice increases. (TIF) [file pcbi.1010306.s022.tif]

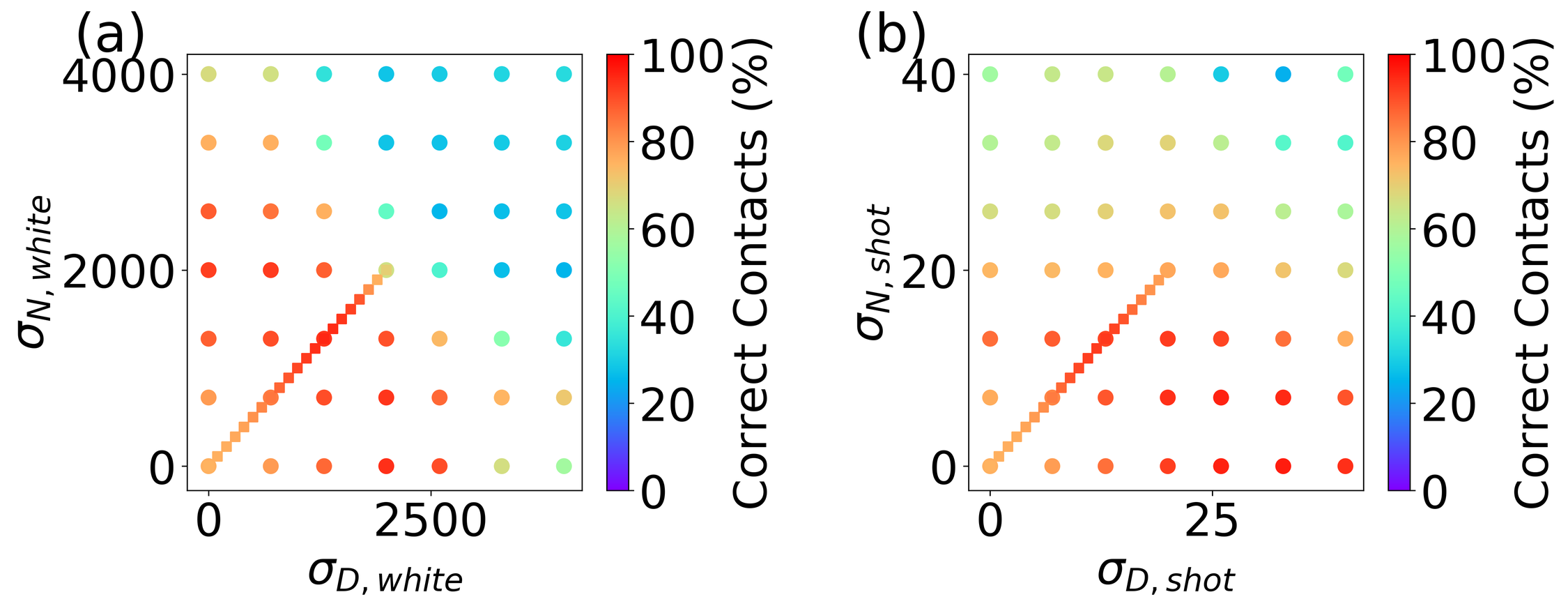

Supplement: S20 Fig — The correct contacts are an averaged after a system has relaxed for 1000 hr. (a) The percent of correct contacts as the level of white noise added to cellular Notch (y) and Delta (x) changes. A slope of one is consistent with results from the main text. The results are nearly symmetric with higher noise on Delta correlated with slightly more ordered patterns at high noise levels. (b) Same as (a) for shot noise. The results are not as symmetric in this case with higher levels of noise on Delta correlated with greater ordered compared to the same level of noise on Notch. The results are averaged over 20 independent simulations starting from different randomized initial conditions after letting the system relax for 1000hr. (TIF) [file pcbi.1010306.s023.tif]

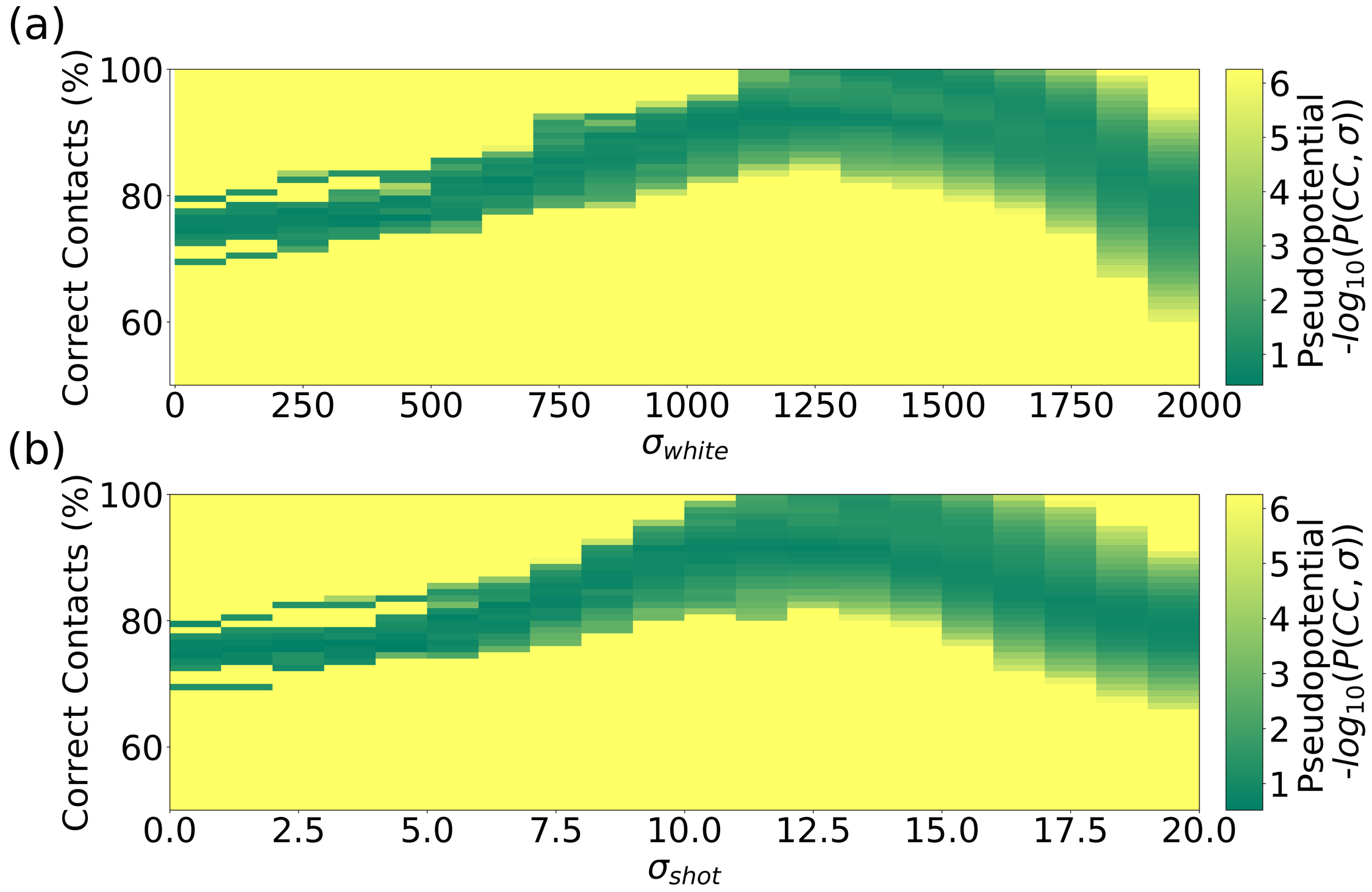

Supplement: S21 Fig — (a) The pseudopotential landscape U = -log10 P(σ,CC) as a function of correct contacts fraction and white noise amplitude (σwhite). (b) Same as (a) but for shot noise amplitude (σshot). The landscapes were constructed using data from 20 independent simulations. (TIF) [file pcbi.1010306.s024.tif]

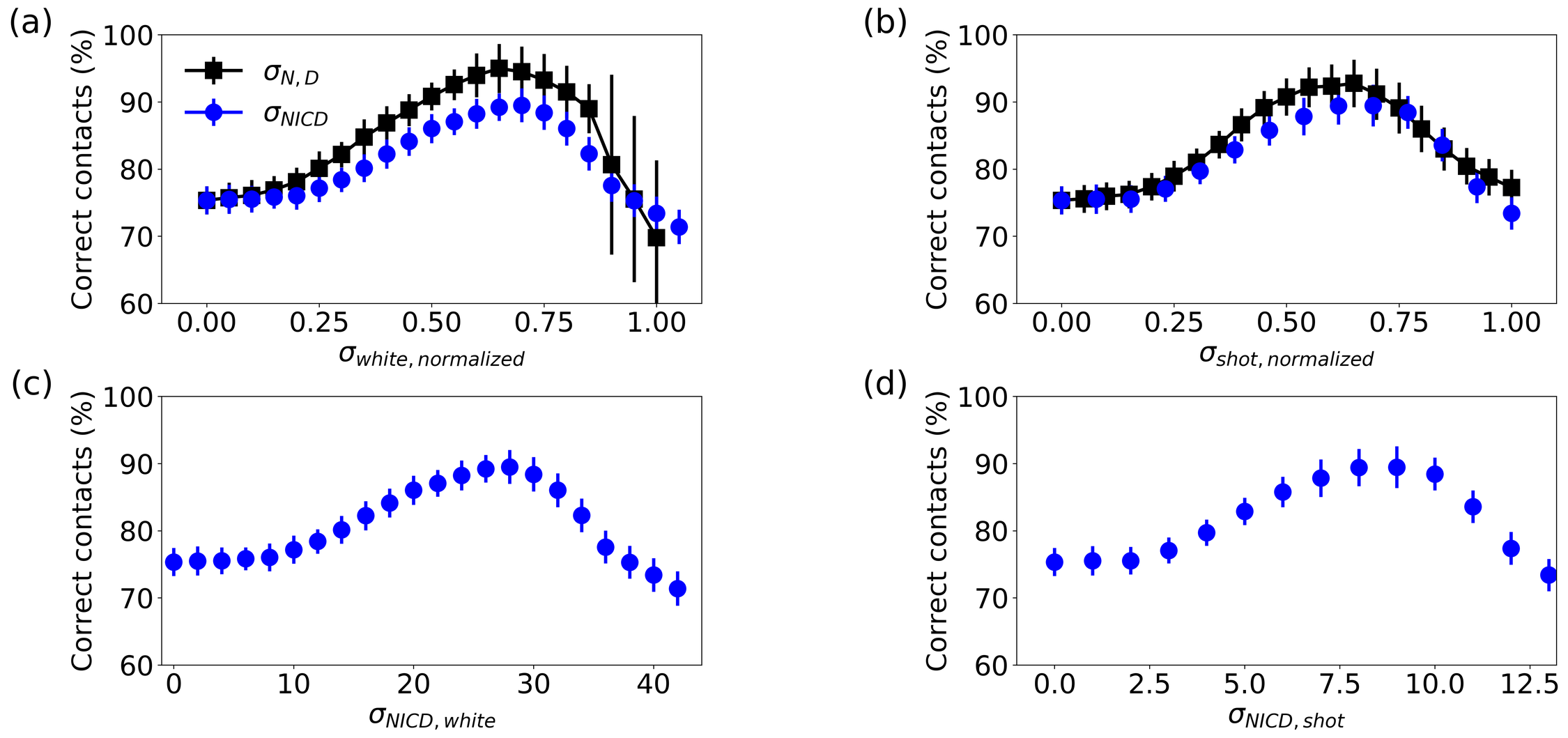

Supplement: S22 Fig — (a) The percent of correct contacts when noise is on Notch and Delta (black) compared to when noise is only included on NICD (blue). The x-axis is normalized such that σwhite,normalized = σwhite/200 (for noise on Notch and Delta) and σwhite,normalized = σNICD,white/40 (for noise on NICD). (b) Same as (a) but the x-axis is normalized such that σshot,normalized = σshot/20 (for noise on Notch and Delta) and σshot,normalized = σNICD,shot/13 (for noise on NICD). (c) The percent of correct contacts when noise is only included on NICD as a function of the white noise amplitudes σNICD.white. (d) Same as (c) except for shot noise. The results for both models (noise on N and D or noise on NICD) are averaged over 20 simulations after 1000hr of relaxation. (TIF) [file pcbi.1010306.s025.tif]

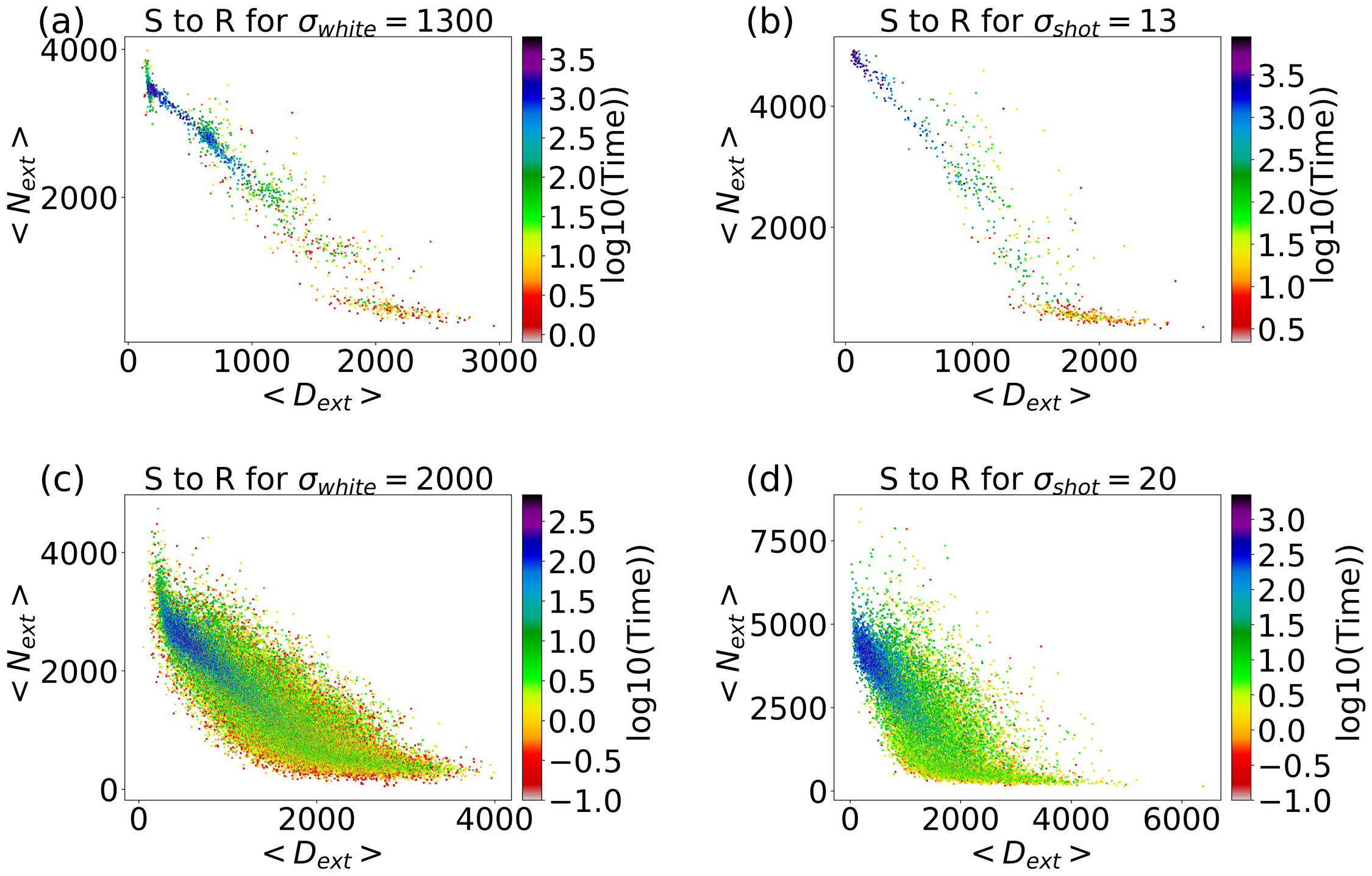

Supplement: S23 Fig — (a) The average transition time for a Sender cell as a function of the Notch and Delta of its neighboring cells for σwhite = 1300. (b) Same as (a) but for σshot = 13. (c) Same as (a) but for σwhite = 2000. (d) Same as (a) but for σshot = 20. The simulations start with randomized initial conditions and the simulation is allowed to relax for 1000 hr. The times are averaged over 9000 hr of a single simulation and all cells in the lattice as a function of the Notch and Delta of the neighboring cells (NEXT and DEXT). (TIF) [file pcbi.1010306.s026.tif]

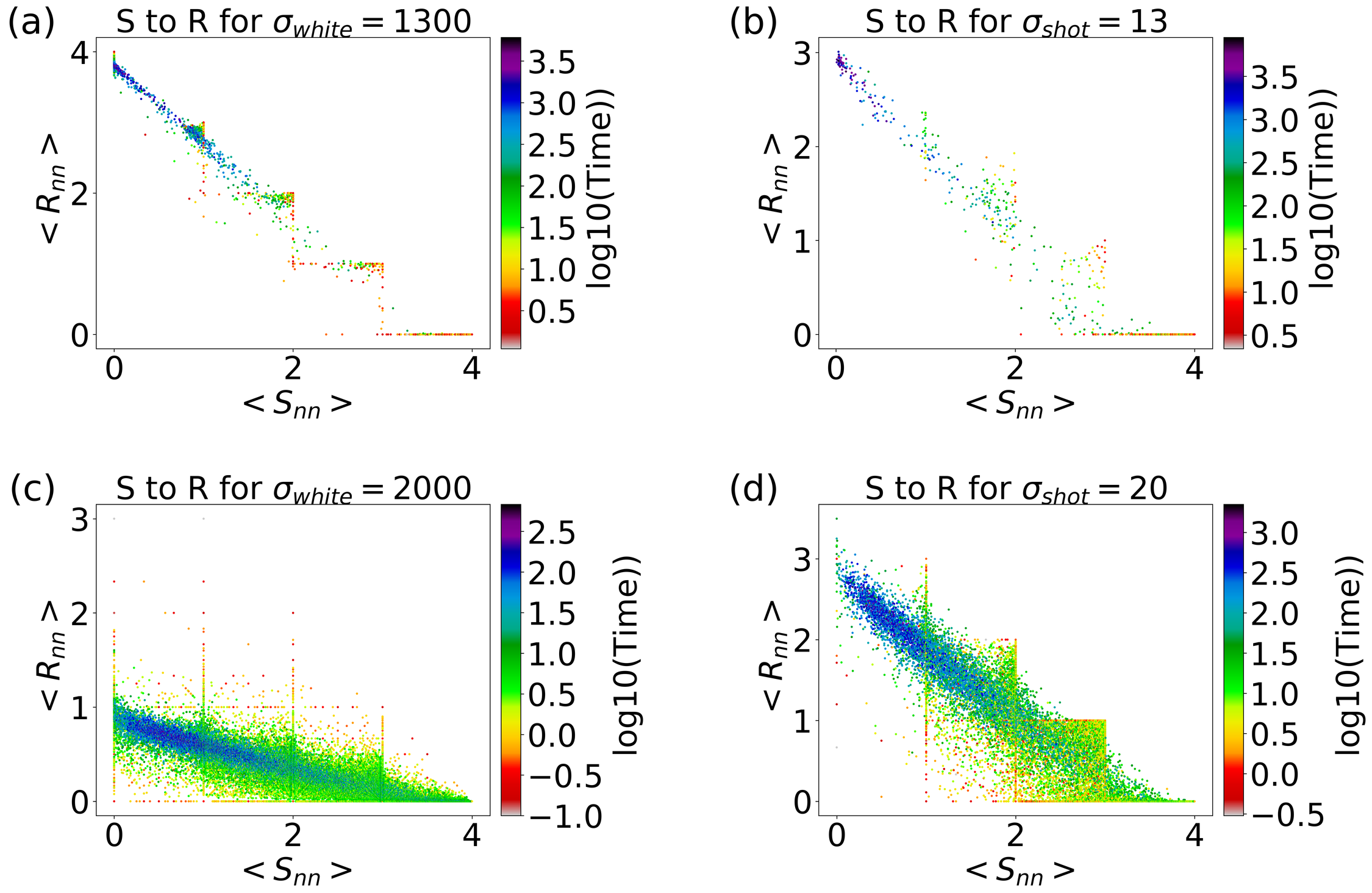

Supplement: S24 Fig — (a) The average transition time as a function of the nearest neighbors that are Senders () and Receivers () for σwhite = 1300. (b) Same as (a) for σshot = 13. (c) same as (a) for σwhite = 2000. (d) Same as (a) for σshot = 20. The simulation is started with randomized initial lattices and relax for 1000 hr. The results are the average of all cells over 9000 hr of a single simulation. (TIF) [file pcbi.1010306.s027.tif]

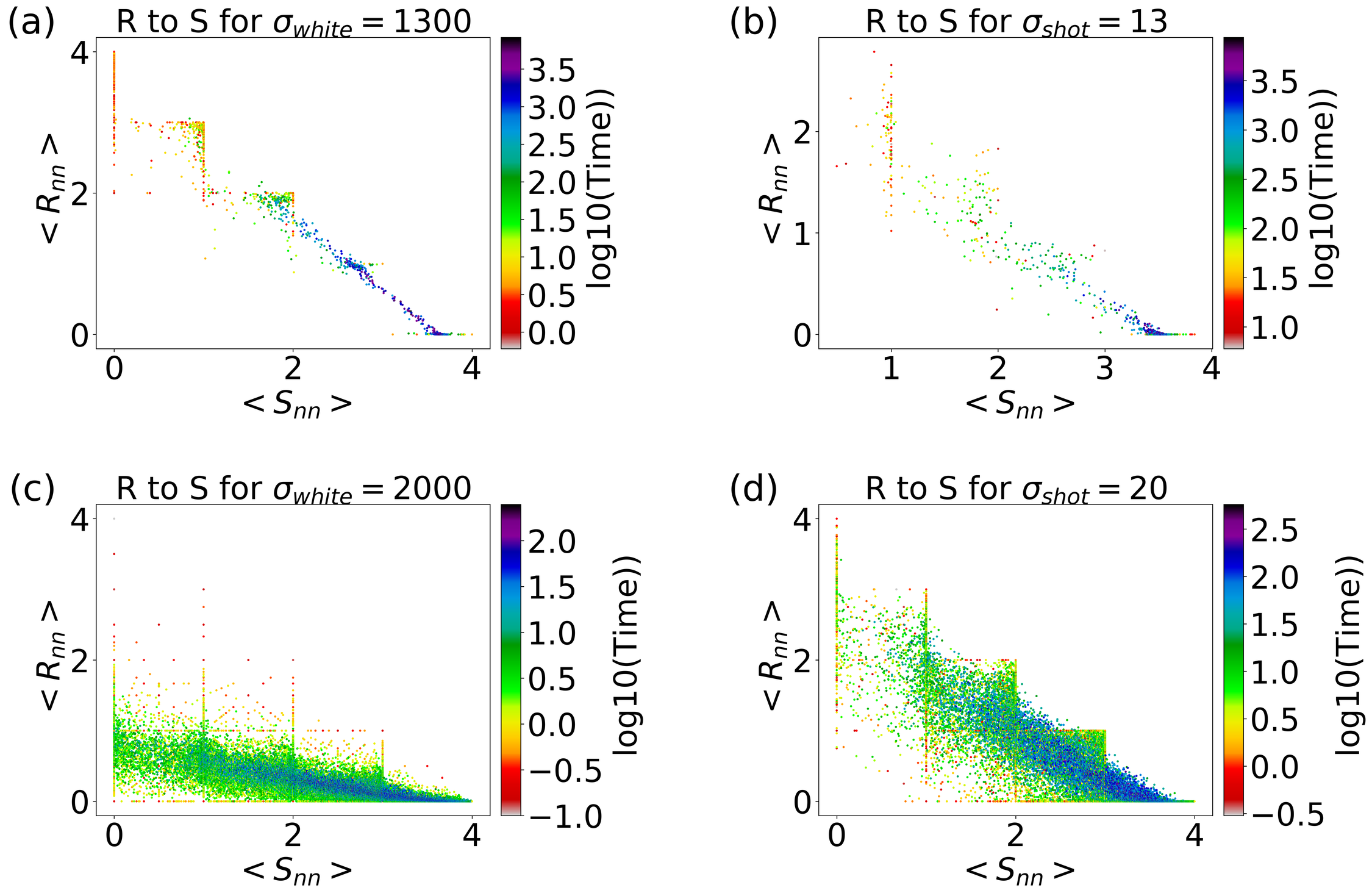

Supplement: S25 Fig — (a) The average transition time as a function of the nearest neighbors that are Senders () and Receivers () for σwhite = 1300. (b) Same as (a) for σshot = 13. (c) same as (a) for σwhite = 2000. (d) Same as (a) for σshot = 20. The simulation is started with randomized initial lattices and relax for 1000 hr. The results are the average of all cells over 9000 hr of a single simulation. (TIF) [file pcbi.1010306.s028.tif]

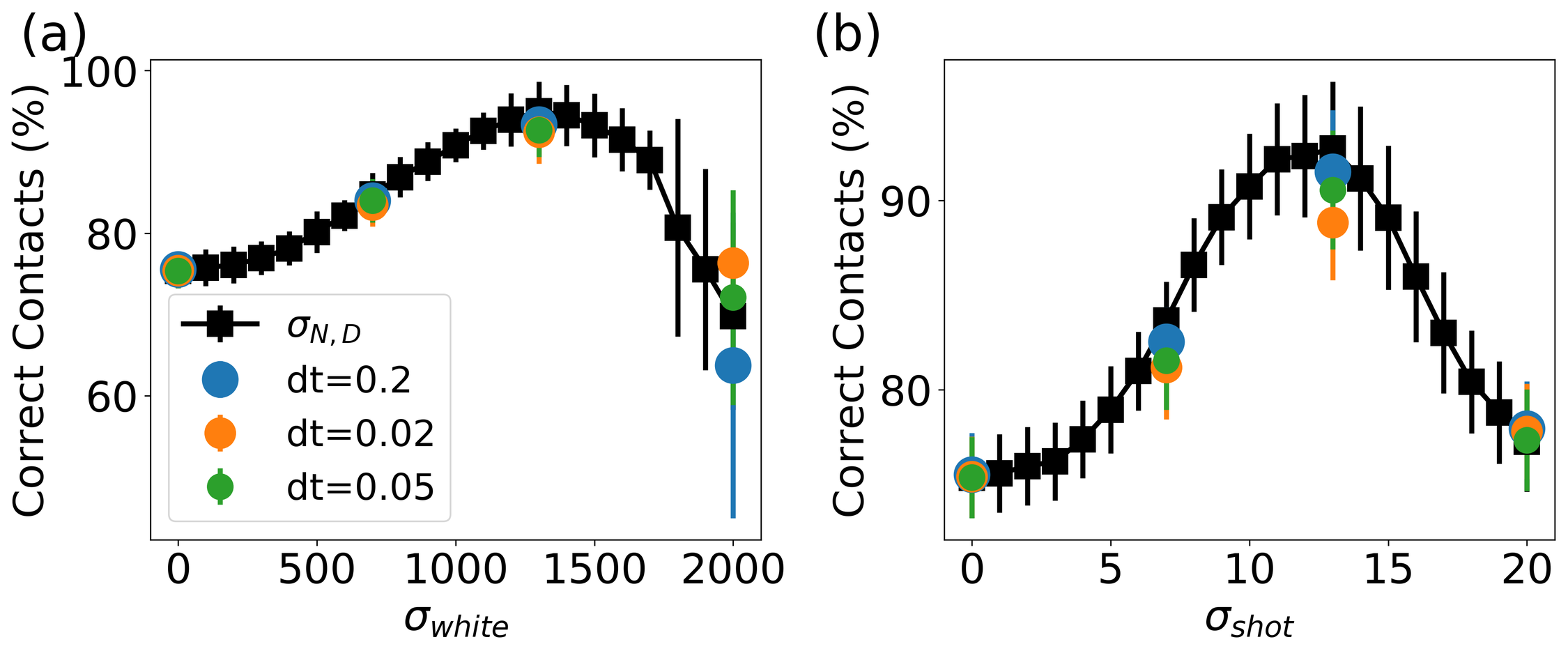

Supplement: S26 Fig — (a) The percent of correct contacts when white noise is present in the system with different time steps of 0.05 hr (green), 0.02 hr (orange), 0.2 hr (blue), and 0.1 hr (black, value used in main results). (b) Same as (a) but for shot noise. These results, especially for the zero noise case, show that the Euler method can be used for our results and a time step of dt = 0.1 hr is sufficient. The results are averaged over the last half of the simulation for 20 independent simulations of a 16x16 square multicell layer. (TIF) [file pcbi.1010306.s029.tif]

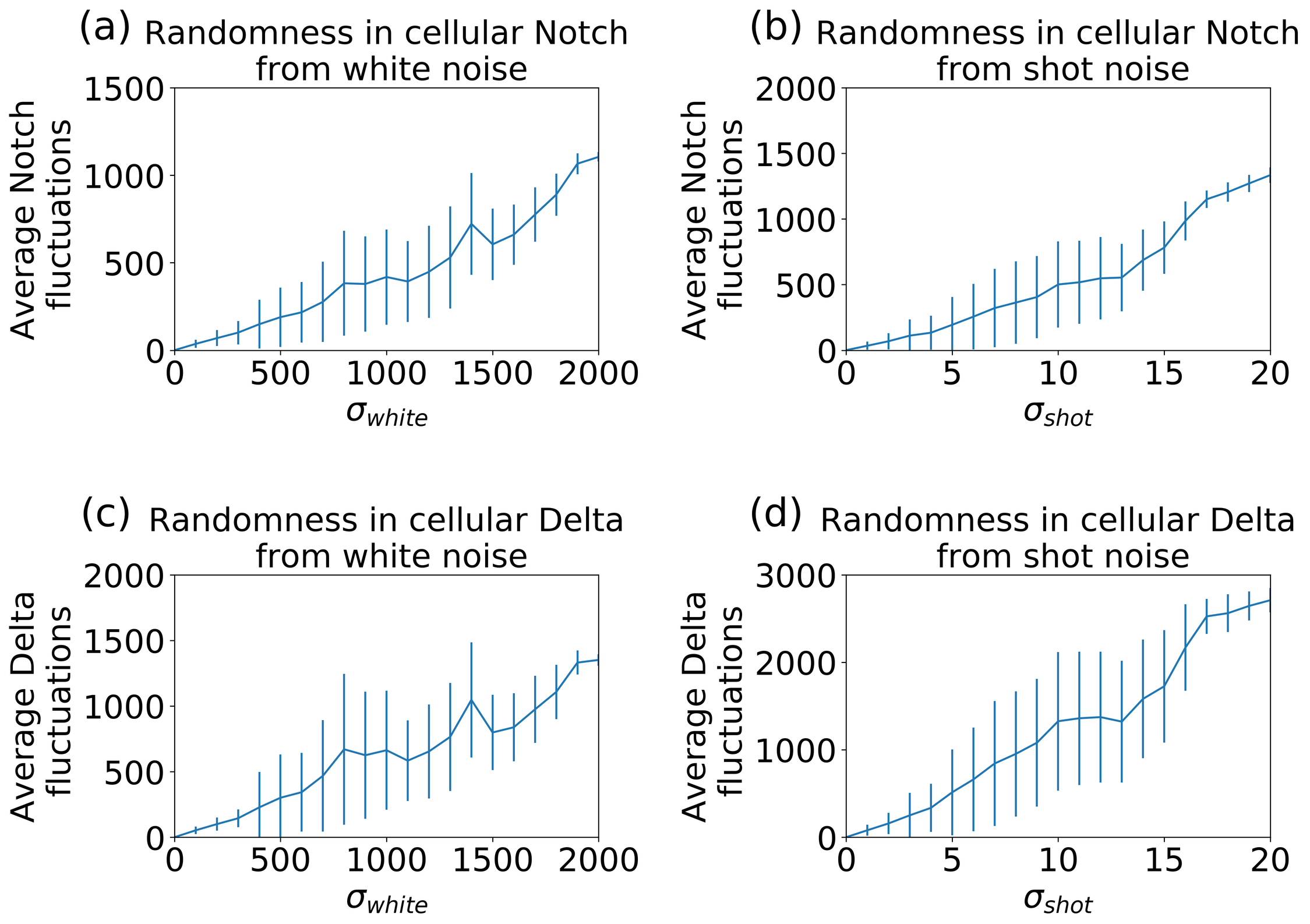

Supplement: S27 Fig — (a) The randomness in the cellular level of notch when white noise is present. The standard deviation of the cellular Notch level was computed for every cell once the system relaxed for 1000 hr. The average value of this standard deviation is plotted showing an increase of Notch fluctuations as white noise levels increased which confirms randomness has been implemented in the system. (b) The same as (a) but for shot noise. (c) The same as (a) but for cellular Delta concentrations. (d) The same as (c) but for shot noise. (TIF) [file pcbi.1010306.s030.tif]
